# Assessment of immunity to polio among Rohingya children in Cox’s Bazar, Bangladesh, 2018: A cross-sectional survey

**DOI:** 10.1371/journal.pmed.1003070

**Published:** 2020-03-31

**Authors:** Concepcion F. Estivariz, Sarah D. Bennett, Jacquelyn S. Lickness, Leora R. Feldstein, William C. Weldon, Eva Leidman, Daniel C. Ehlman, Muhammad F. H. Khan, Jucy M. Adhikari, Mainul Hasan, Mallick M. Billah, M. Steven Oberste, A. S. M. Alamgir, Meerjady D. Flora

**Affiliations:** 1 Global Immunization Division, Centers for Disease Control and Prevention, Atlanta, Georgia, United States of America; 2 Epidemic Intelligence Service, Centers for Disease Control and Prevention, Atlanta, Georgia, United States of America; 3 Division of Viral Diseases, Centers for Disease Control and Prevention, Atlanta, Georgia, United States of America; 4 Division of Global Health Protection, Centers for Disease Control and Prevention, Atlanta, Georgia, United States of America; 5 World Health Organization, Dhaka, Bangladesh; 6 United Nations Children’s Fund, Dhaka, Bangladesh; 7 Institute of Epidemiology, Disease Control and Research, Dhaka, Bangladesh; Johns Hopkins University Bloomberg School of Public Health, UNITED STATES

## Abstract

**Background:**

We performed a cross-sectional survey in April–May 2018 among Rohingya in Cox’s Bazar, Bangladesh, to assess polio immunity and inform vaccination strategies.

**Methods and findings:**

Rohingya children aged 1–6 years (younger group) and 7–14 years (older group) were selected using multi-stage cluster sampling in makeshift settlements and simple random sampling in Nayapara registered camp. Surveyors asked parents/caregivers if the child received any oral poliovirus vaccine (OPV) in Myanmar and, for younger children, if the child received vaccine in any of the 5 campaigns delivering bivalent OPV (serotypes 1 and 3) conducted during September 2017–April 2018 in Cox’s Bazar. Dried blood spot (DBS) specimens were tested for neutralizing antibodies to poliovirus types 1, 2, and 3 in 580 younger and 297 older children. Titers ≥ 1:8 were considered protective. Among 632 children (335 aged 1–6 years, 297 aged 7–14 years) enrolled in the study in makeshift settlements, 51% were male and 89% had arrived after August 9, 2017. Among 245 children (all aged 1–6 years) enrolled in the study in Nayapara, 54% were male and 10% had arrived after August 9, 2017. Among younger children, 74% in makeshift settlements and 92% in Nayapara received >3 bivalent OPV doses in campaigns. Type 1 seroprevalence was 85% (95% CI 80%–89%) among younger children and 91% (95% CI 86%–95%) among older children in makeshift settlements, and 92% (88%–95%) among younger children in Nayapara. Type 2 seroprevalence was lower among younger children than older children in makeshift settlements (74% [95% CI 68%–79%] versus 97% [95% CI 94%–99%], *p <* 0.001), and was 69% (95% CI 63%–74%) among younger children in Nayapara. Type 3 seroprevalence was below 75% for both age groups and areas. The limitations of this study are unknown routine immunization history and poor retention of vaccination cards.

**Conclusions:**

Younger Rohingya children had immunity gaps to all 3 polio serotypes and should be targeted by future campaigns and catch-up routine immunization. DBS collection can enhance the reliability of assessments of outbreak risk and vaccination strategy impact in emergency settings.

## Abstract 2

Concepción Fernandez Estívariz et al report that Rohingya children aged 1-6 years have immunity gaps to all polio serotypes and should be targeted by future campaigns for catch up immunisation.

## Author summary

**Why was this study done?:**

- Between August 2017 and January 2018, almost 700,000 Rohingya people arrived in Cox’s Bazar District, Bangladesh, and settled in 2 refugee camps and in makeshift settlements around the camps. Crowding and inadequate access to safe water, sanitation, and healthcare facilitated outbreaks of infectious diseases that spread to the surrounding community.
- Ongoing outbreaks of measles and diphtheria in May 2018, despite several vaccination campaigns, suggested that some children remained unprotected for vaccine-preventable diseases.
- We conducted this survey to evaluate immunity against poliovirus and guide new vaccination activities.

**What did the researchers do and find?:**

- We conducted a cross-sectional survey among Rohingya children 1–14 years of age during April–May 2018 that included an interview about vaccines received in recent campaigns and collection of blood samples through finger prick to test for antibodies against poliovirus.
- Protection against poliovirus type 1 was lower among children aged below 7 years than in older children in the makeshift settlements, despite most children having received several doses of oral polio vaccine in campaigns.
- Protection against all poliovirus was lowest in children below 3 years of age.

**What do these findings mean?:**

- These findings suggested that children below 7 years of age recently arrived in the camp/settlements had immunity gaps after the vaccination campaigns that increased the risk of polio outbreaks.
- Based upon this information, we recommended to the agencies that conducted the campaigns that they include polio vaccines in future campaigns targeting only children below 5 years of age.
- Rapid scale-up of routine immunization services would be necessary to close immunity gaps among children below 2 years of age.
- Collection of dried blood specimens through finger prick for antibody testing is feasible in emergency settings, and provides crucial information for assessment of outbreak risk and impact of outbreak response strategies.

## Introduction

Between August 2017 and January 2018, almost 700,000 ethnic minority Rohingya fleeing violence in Myanmar arrived in Cox’s Bazar District, Bangladesh. They joined approximately 200,000 Rohingya already settled in 2 registered refugee camps, Kutupalong and Nayapara, and within the Bangladeshi community [1]. The new arrivals set up residence in crowded makeshift settlements around the existing camps with inadequate access to safe water, sanitation, and healthcare, which increased their vulnerability to infectious diseases. Cases of measles and diphtheria, in addition to increased diarrhea and respiratory infections, were reported during September–November 2017 [2]. Although the outbreaks spilled over to the host community, unregistered recent arrivals appeared to have higher morbidity [3]. To control ongoing outbreaks and prevent new ones, the Bangladesh Ministry of Health and Family Welfare and international partners conducted 7 immunization campaigns between September 2017 and April 2018, targeting different age groups with a variety of antigens [1].

Trivalent oral poliovirus vaccine (tOPV, which contains serotypes 1, 2, and 3) was withdrawn from global use in April 2016 to reduce the burden of type 2 circulating vaccine-derived poliovirus (cVDPV) outbreaks [4]. Following tOPV withdrawal, primary immunization against polio included 3 doses of bivalent oral poliovirus vaccine (bOPV, which contains serotypes 1 and 3) administered at 6, 10, and 14 weeks of age in Bangladesh and at 2, 4, and 6 months of age in Myanmar, plus 1 dose of inactivated poliovirus vaccine (IPV) at 14 weeks (Bangladesh) or 4 months (Myanmar) to provide protection for type 2 polio [5–7]. bOPV also became the vaccine of choice for preventative or outbreak response campaigns, whereas monovalent oral poliovirus vaccine (OPV) type 2 was restricted for use in type 2 cVDPV outbreaks [4].

In Cox’s Bazar, bOPV was included in 5 immunization campaigns, targeting children below 5 or 7 years of age (depending on the campaign), because of the potential risks of transmission of wild poliovirus following importation from endemic countries and the emergence of cVDPV. Outbreaks of paralytic polio caused by cVDPV usually emerge in settings with low coverage with OPV and in settings with factors that favor poliovirus transmission, such as crowding and suboptimal sanitation [8,9]. Myanmar experienced cVDPV emergences in 2006–2007 (type 1, 7 cases) [10], 2012 (type 1, 1 case), and 2015 (type 2, 2 cases) [11], with the 2015 outbreak affecting Rohingya communities in Rakhine State. Although vaccination campaigns with tOPV were conducted in Myanmar between 2007 and 2016 to control the outbreaks, some campaigns were small and targeted only certain areas [7], such that the immunity of the Rohingya children recently arrived in Cox’s Bazar was uncertain.

Following the vaccination campaigns and other outbreak control measures in Cox’s Bazar, the number of measles and diphtheria cases detected in the camps decreased, but transmission persisted through May 2018 [1]. To evaluate remaining immunity gaps and direct future immunization activities, we conducted a cross-sectional immunity assessment and vaccination coverage survey among refugee children aged 1 to 14 years. This report presents the results for polio, and discusses lessons learned with the use of dried blood spot (DBS)–based serosurveys to support polio eradication strategies. Seroprevalence testing for other preventable diseases was done with different methodologies and with a different final sample set; those data will be presented in a related paper.

## Methods

This study is reported as per the Strengthening the Reporting of Observational Studies in Epidemiology (STROBE) guideline (S1 STROBE Checklist). The methodology and analysis plan followed those described in the study protocol (S1 Protocol).

### Study design

This was a cross-sectional study among children 1–14 years of age, living in makeshift settlements or in the Nayapara registered refugee camp in Cox’s Bazar, Bangladesh, April 28–May 28, 2018. Children aged 1–6 years and 7–14 years were treated as independent populations because only the younger age group could have received bOPV in the recent campaigns. Polio immunity among children in the older age group (7–14 years) would depend on tOPV doses received through routine immunization or campaigns conducted in Myanmar following cVDPV outbreaks [7]. The makeshift settlements and registered camp were treated as independent populations because access to health services, including immunization, was expected to be different between residents in makeshift settlements and the registered camp [12].

We applied random sampling in Nayapara, where detailed household information was available, and cluster sampling in the makeshift settlements. Because the vaccination survey was conducted in coordination with an Emergency Nutrition Assessment, sampling selection and size estimations were adapted to the requirements of both surveys [13]. In the Nayapara refugee camp, the United Nations High Commissioner for Refugees randomly selected 411 registered households and 113 unregistered households, with the ratio of registered to unregistered households proportional to the ratio observed in the population of the camp. The makeshift settlements were divided into blocks and further into sub-blocks (mean size 108 households, range 26–970), from which we selected 55 sub-blocks (clusters) by population proportional to size, using ENA software [13]. Within each sampled cluster, surveyors enumerated all the households about 1 week before data collection, and selected 13 households by simple random sampling using a random number generator. Survey teams visited all 13 households within 1 day in the makeshift settlements, and 8 households per day in Nayapara to prevent data collectors observing Ramadan from overexertion. Within each household 1 child aged 6 months–6 years and 1 child aged 7–14 years were randomly selected, if applicable. Efforts were made to revisit absent households at least twice. There was no replacement for clusters, households, or children.

The final sample required for the nutrition survey was 715 households in the makeshift settlements (55 clusters with 13 households each) and 524 households in Nayapara. Based upon a survey conducted in November 2017 [12], we estimated that 60%–65% of households would have 1 child aged 1–14 years, and non-response would be 15%–30%. Therefore, we expected to enroll 367 and 226 children aged 1–6 years in the makeshift settlements and Nayapara, respectively, and 326 children aged 7–14 years in the makeshift settlements. Assuming a design effect of 1.5 for the makeshift settlements and 1 for Nayapara, our estimated precision was ±6%–7% for a seroprevalence of 50%. Specimen collection among children aged 7–14 years could not be done in Nayapara because the survey there took place during Ramadan fasting. The Institutional Review Board of the Institute of Epidemiology, Disease Control and Research of Bangladesh had not approved specimen collection among older children during Ramadan.

### Vaccination coverage survey

For each child 6 months to 14 years of age enrolled, surveyors collected demographic information (sex, age, date of arrival to the camp/settlements) and a vaccination history. Information was collected on electronic tablets using KoBoCollect software (version 1.4.8.), which allowed daily upload and remote monitoring of data quality. The questionnaire was standardized in English and Bangla languages because the Rohingya language only exists in oral form (S1 Vaccination Questionnaire). Surveyors spoke Chittagonian, which is similar to Rohingya, and translated the questions during the interview, using a vocabulary agreed upon during surveyor training. Vaccination questions included whether the child had received any injectable or oral poliovirus vaccine in Myanmar or at the border upon entry, and whether the child had received vaccine in each of the 7 campaigns conducted in the camps/settlements between September 2017 and April 2018. Vaccination cards for routine immunization and campaigns were checked, and parent/caregiver recall used when vaccination cards were not available.

Most campaigns included several antigens, and we assumed that if the parent/caregiver reported that the child had received vaccine in a certain campaign, he or she would have received all the antigens recommended for that age group. During the September 2018 campaign, which delivered bOPV to children below 5 years of age and measles/rubella vaccine to children aged 9 months to 15 years, we assumed that children who were aged around 5 or 6 years received both vaccines, because age estimations were imprecise and under such conditions older children are often vaccinated in campaigns.

### Specimen collection and testing

Surveyors drew blood from children aged 1–14 years by finger prick, using a retractable safety lancet, and collected 3 or 4 drops on a Whatman 903 Protein Saver Card. DBS samples were not collected from enrolled children aged 6–11 months. Immediately after specimen collection, cards were placed on racks within a plastic box to allow for air-drying during house-to-house visits. At the end of each day, the cards were transferred to individual sealed plastic bags with desiccant gels and a humidity indicator card. Samples were stored at room temperature for up to 2 weeks, before transportation to the US Centers for Disease Control and Prevention in Atlanta. On arrival to the laboratory, the DBS samples were logged, randomized, and stored at −20°C until testing.

For testing, 3 6-mm punches (1 per serotype) were eluted to extract about 6 μl of serum per punch. Testing for neutralizing antibodies against poliovirus types 1, 2, and 3 was performed using a modified poliovirus microneutralization assay [14]. Each test was run in triplicate with 2-fold serial dilutions ranging from 1:8 to 1:1,024. A neutralizing antibody titer of 1:8 has been shown to correlate with protection from poliomyelitis and was used as the threshold for seropositivity [14].

### Statistical analysis

Demographic characteristics and vaccination history of participants with a DBS sample were summarized. Seroprevalence and 95% (Wilson) confidence limits were estimated for each serotype, age group, and camp/settlements. We evaluated the potential effect of factors such as sex, age, number of OPV doses received in recent campaigns, and receipt of any OPV in Myanmar on seroprevalence using Fisher’s test or Rao–Scott chi-squared test. For a subset of children aged 1–4 years for whom nutritional status was assessed [12,15], we also evaluated the potential effect of acute malnutrition (low weight for height, or wasting) or chronic malnutrition (low height for age, or stunting) using Rao–Scott chi-squared test [15].

The analysis was adjusted to account for the cluster design and sampling weights in makeshift settlements. A *p*-value < 0.05 was considered significant. Analysis were performed using SAS version 9.3 [16]; R version 3.2.3 was used for the creation of graphics [17].

### Ethical approval

The survey protocol and script for verbal consent were approved by the Institutional Review Board of the Institute of Epidemiology, Disease Control and Research, of Bangladesh. The US Centers for Disease Control and Prevention considered the survey an epidemic disease control activity. Before child enrollment, surveyors explained the objectives, risks, and benefits of participation in the study to the parent/caregiver and obtained the parent/caregiver’s verbal informed consent in the Rohingya language. After the parent/caregiver had provided verbal consent, surveyors conducted study procedures, although some children refused specimen collection after caregiver’s consent.

## Results

### Recruitment and final study sample

**Fig 1:**
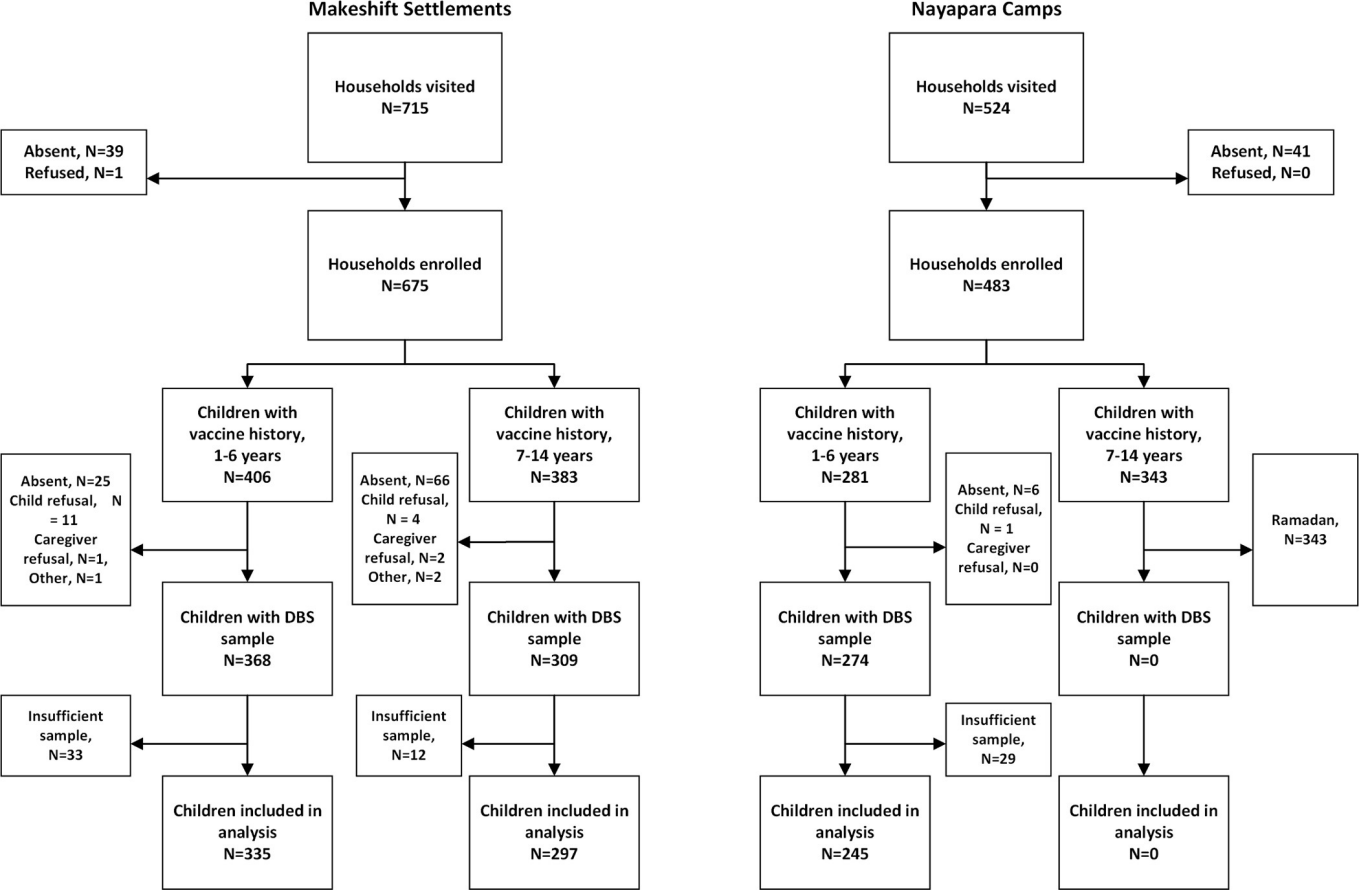
Sample selection among Rohingya children in Cox’s Bazar, Bangladesh, April–May 2018. DBS, dried blood spot.

The survey was conducted during April 28–May 9, 2018, in the makeshift settlements and during May 17–28, 2018, in Nayapara. As shown in Fig 1, families were available during the survey visits in 94% and 92% of the houses visited in the makeshift settlements and Nayapara, respectively. All except 1 parent/caregiver accepted participating in the vaccination survey. In the makeshift settlements, DBS collection and testing were possible for 335 (83%) candidate children aged 1–6 years and 297 (78%) candidate children aged 7–14 years. In Nayapara, DBS testing results were available for 245 (87%) participants aged 1–6 years (Fig 1). The most common reason for not having polio immunity data among older children in the makeshift settlements was that a DBS sample was not collected because the child was absent (66/86, 77%), and the most common reason among younger children in both areas was collection of insufficient specimen for testing (62/107, 58%).

### Characteristics of the population surveyed

The demographic characteristics and vaccination history of study participants with a DBS sample tested for poliovirus immunity showed some differences in potential access to vaccines (Table 1). Most children living in the makeshift settlements (89%) had arrived after August 9, 2017. In Nayapara, only 10% of children had arrived with the recent influx, and 89% of younger children were born in the camp.

**Table 1:** Demographic and vaccination history of Rohingya children with DBS sample available, Cox’s Bazar, Bangladesh, April–May 2018.

Characteristic	Makeshift settlements, 1–6 years of age			Makeshift settlements, 7–14 years of age			Nayapara, 1–6 years of age		
	*n*	*N*	Median (IQR) or percent	*n*	*N*	Median (IQR) or percent	*n*	*N*	Median (IQR) or percent
Epidemiology and prior vaccination									
Household size		335	5 (4–7)		297	6 (4–8)		245	6 (4–7)
Registered refugee	0	335	0	0	297	0	216	245	88
Sex male	171	335	51	152	297	51	133	245	54
Arrival after August 9, 2017	298	335	89	264	297	89	24	245	10
Born in the camp/settlements*	11	329	3	10	290	4	217	244	89
Received any OPV in Myanmar	239	320	75	234	283	83	19	26	73
Received bOPV at border	9	324	3	14	286	5	1	28	4
bOPV doses received in campaigns in Cox’s Bazar									
Doses		335	4 (3–5)		—	—		244	5 (4–5)
0 doses	11	335	3	—	—	—	4	244	2
1–3 doses	77	335	23	—	—	—	15	244	6
4–5 doses	247	335	74	—	—	—	225	244	92
Participation in campaigns distributing bOPV in Cox’s Bazar									
MR first round—September 2017	250	330	76	198	294	67	212	240	88
OCV second round—November 2017	253	326	78	—	—	—	219	241	91
Penta first round—January 2018	274	325	84	240	294	82	222	243	91
Penta second round—February 2018	288	327	88	253	294	86	231	242	95
Penta third round—March 2018	269	330	82	245	294	83	228	240	95

bOPV, bivalent oral poliovirus vaccine; DBS, dried blood spot; MR, measles/rubella vaccine; OCV, oral cholera vaccine; OPV, oral poliovirus vaccine; Penta, pentavalent vaccine containing diphtheria, tetanus toxoid, pertussis, *Haemophilus influenzae*, and hepatitis B antigens.

*For children in makeshift settlements, this information was estimated from date of family arrival and reported age.

During the campaigns conducted in Cox’s Bazar, 74% of younger children in the makeshift settlements and 92% of younger children in Nayapara had received >3 bOPV doses (Table 1). Documentation by vaccination card was available for only 12% of doses reportedly received. Older children were not eligible for bOPV in any campaign. A high proportion of children in the makeshift settlements (75%–83%) reported having received some OPV in Myanmar, but we did not ask for the number of doses (Table 1). Among 40 children aged 6–11 months for whom we did not collect DBS samples, 45% had received >3 bOPV doses during the campaigns, and 14% had received some OPV in Myanmar.

### Seroprevalence and antibody titers

Type 1 seroprevalence was 85% (95% CI 80%–89%) and 91% (95% CI 86%–95%) among the younger and older age groups in the makeshift settlements, respectively (*p =* 0.07). In Nayapara, seroprevalence among younger children was 92% (95% CI 88%–95%) (Table 2). Type 2 seroprevalence was lower among younger than older children living in the makeshift settlements (74% [95% CI 68%–93%] versus 97% [95% CI 94%–99%], *p <* 0.001), and was also low among younger children in Nayapara (69% [95% CI 63%–74%]). Type 3 seroprevalence was below 75%, and similar for both age groups and locations. Median and interquartile range of antibody titers in seropositive children are shown on Table 2.

**Table 2:** Seroprevalence and neutralizing antibody titers to polio by camp/settlements and age group—Rohingya children, Cox’s Bazar, Bangladesh, April–May 2018.

Outcome	Makeshift settlements, 1–6 years of age		Makeshift settlements, 7–14 years of age		Nayapara, 1–6 years of age	
	*n/N* or *N*	Percent (95% CI) or median (IQR)	*n/N* or *N*	Percent (95% CI) or median (IQR)	*n/N* or *N*	Percent (95% CI) or median (IQR)
Polio seropositive						
Type 1	290/335	85 (80, 89)	275/297	91 (86, 95)	225/245	92 (88, 95)
Type 2	252/335	74 (68, 79)	285/297	97 (94, 99)	169/245	69 (63, 74)
Type 3	239/335	72 (67, 77)	227/297	74 (68, 80)	179/245	73 (67, 78)
Polio neutralizing antibody titers (among seropositive)						
Type 1	290	90 (25, 619)	275	60 (20, 270)	225	72 (23, 455)
Type 2	252	101 (22, 233)	285	50 (21, 146)	169	36 (18, 91)
Type 3	239	48 (14, 183)	227	32 (14, 102)	179	45 (18, 144)

Data for seroprevalence are presented as percent seropositive and Wilson 95% confidence intervals. Reciprocal antibody titers are presented as median and interquartile range.

### Assessment of risk factors for seroprevalence

**Fig 2:**
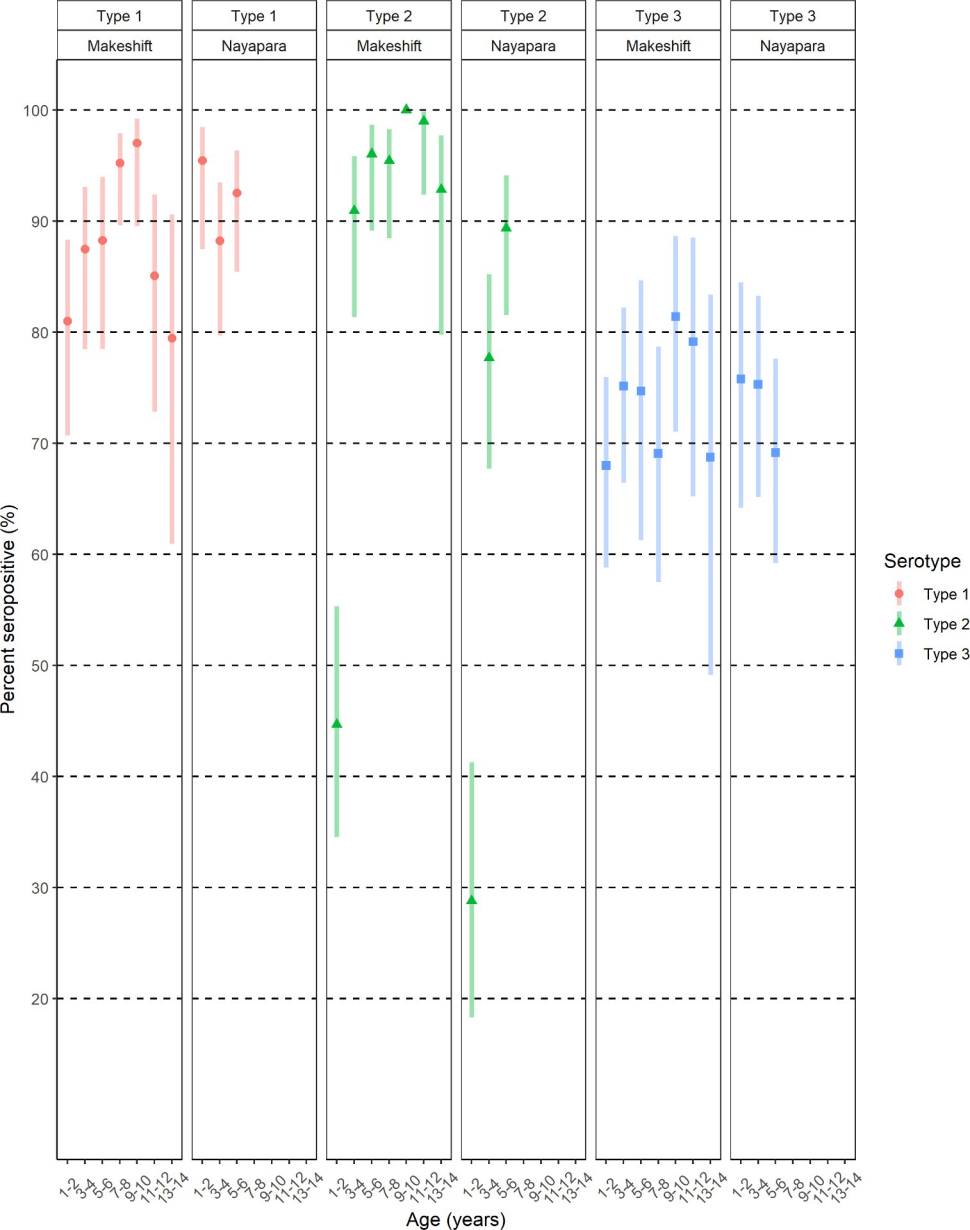
Seroprevalence to poliovirus by serotype, camp/settlements, and age (2-year intervals)—Rohingya children in Cox’s Bazar, Bangladesh, April–May 2018. Data presented as percent seropositive and Wilson 95% confidence intervals.

Univariate analysis found some influence of age in immunity to polio types 1 and 2. For type 1, the proportion of seropositive children in the makeshift settlements tended to be lower among children aged 1–2 years than among those aged 9–10 years (81% [95% CI 71%–88%] versus 97% [95% CI 90%–99%]), but we did not detect a significant correlation between age and seroprevalence (*p =* 0.06; Fig 2 and Table 3). Type 2 seroprevalence among children in the makeshift settlements was lowest among children aged 1–2 years (45% [95% CI 35%–55%]); within this age group, children aged below 2 years were born after the global switch from tOPV to bOPV in April 2016. Seroprevalence increased to 91% (95% CI 81%–96%) among children aged 3–4 years and 96% (95% CI 89%–99%) among those aged 5–6 years (*p <* 0.001 for trend; Table 3). The same pattern was observed in Nayapara, where only 29% (95% CI 19%–41%) of children aged 1–2 years were seropositive to type 2 poliovirus, compared with 78% (95% CI 68%–85%) among those aged 3–4 years and 89% (95% CI 82%–94%) among those aged 5–6 years (*p <* 0.001 for trend). Type 2 seroprevalence among children aged 7–12 years was above 90% in all age subgroups.

**Table 3:** Seroprevalence to poliovirus by age group, nutritional status, and vaccination history—Rohingya children, Cox’s Bazar, Bangladesh, April–May 2018.

Factor	Makeshift settlements, 1–6 years of age			Makeshift settlements, 7–14 years of age			Nayapara, 1–6 years of age		
	*n/N*	Percent (95% CI)	*p-*Value*	*n/N*	Percent (95% CI)	*p-*Value*	*n/N*	Percent (95% CI)	*p-*Value*
**Type 1**									
**Age (years)**									
1–2	98/120	81 (71–88)	0.4	—	—	—	63/66	96 (88–98)	0.3
3–4	122/137	88 (79–93)		—	—		75/85	88 (88–98)	
5–6	70/78	88 (79–94)		—	—		87/94	93 (85–93)	
7–8	—	—	—	106/114	95 (90–98)	0.06	—	—	—
9–10	—	—		79/81	97 (90–99)		—	—	
11–12	—	—		54/62	85 (73–92)		—	—	
13–14	—	—		36/40	80 (61–91)		—	—	
**bOPV doses received in campaigns in Bangladesh**									
0	7/11	70 (34–91)	0.5	—	—	—	3/4	75 (30–95)	0.1
1–3	65/77	86 (76–93)		—	—		13/15	87 (62–96)	
>3	218/247	86 (80–90)		—	—		208/225	92 (88–95)	
**Received any OPV in Myanmar**									
Yes	211/239	86 (80–90)	0.6	220/234	93 (88–96)	0.2	19/19	100 (83–100)	0.3
No	66/81	83 (69–91)		42/49	83 (67–92)		6/7	86 (49–97)	
Presence of wasting (weight for height below 2 SD)**									
Yes	19/23	89 (70–97)	0.5	—	—	—	18/19	95 (75–99)	1.0
No	201/234	84 (76–89)		—	—		117/129	91 (84–95)	
Presence of stunting (height for age below 2 SD)**									
Yes	82/96	83 (71–91)	0.8	—	—	—	57/60	95 (86–98)	0.2
No	138/161	85 (75–92)		—	—		78/88	89 (80–94)	
**Type 2**									
**Age (years)**									
1–2	53/120	45 (35–55)	<0.001	—	—	—	19/66	29 (19–41)	<0.001
3–4	126/137	91 (81–96)		—	—		66/85	78 (68–85)	
5–6	73/78	96 (89–99)		—	—		84/94	89 (82–94)	
7–8	—	—	—	108/114	95 (88–98)	0.2	—	—	—
9–10	—	—		81/81	100 (—)		—	—	
11–12	—	—		60/62	99 (92–100)		—	—	
13–14	—	—		36/40	93 (80–98)		—	—	
**Received any OPV in Myanmar**									
Yes	190/239	79 (73–84)	0.003	228/234	98 (94–99)	0.3	16/19	84 (62–94)	0.6
No	52/81	60 (48–71)		45/49	94 (83–98)		5/7	71 (37–92)	
Presence of wasting (weight for height below 2 SD)**									
Yes	16/23	74 (53–87)	0.5	—	—	—	7/19	37 (19–59)	0.1
No	163/234	68 (60–74)		—	—		75/129	58 (50–66)	
Presence of stunting (height for age below 2 SD)**									
Yes	67/96	67 (54–78)	0.9	—	—	—	37/60	62 (49–73)	0.2
No	112/161	69 (60–77)		—	—		45/88	51 (41–61)	
**Type 3**									
**Age (years)**									
1–2	79/120	68 (59–76)	0.5	—	—	—	50/66	76 (64–86)	0.6
3–4	102/137	75 (66–82)		—	—		64/85	75 (65–83)	
5–6	58/78	75 (61–85)		—	—		65/94	69 (59–78)	
7–8	—	—	—	85/114	69 (58–79)	0.06	—	—	—
9–10	—	—		65/81	81 (71–89)		—	—	
11–12	—	—		47/62	79 (65–89)		—	—	
13–14	—	—		30/40	69 (49–93)		—	—	
**bOPV doses received in campaigns in Bangladesh**									
0	8/11	81 (52–95)	0.4	—	—	—	4/4	100 (51–100)	0.3
1–3	50/77	66 (54–76)		—	—		9/15	60 (36–80)	
>3	181/247	74 (67–79)		—	—		165/225	73 (67–79)	
**Received any OPV in Myanmar**									
Yes	178/239	74 (68–80)	0.2	154/197	76 (66–83)		13/19	68 (46–85)	0.7
No	51/81	66 (55–76)		73/100	72 (58–82)		4/7	57 (25–84)	
Presence of wasting (weight for height below 2 SD)**									
Yes	18/23	81 (61–92)	0.2	—	—	—	17/19	90 (69–97)	0.2
No	163/234	71 (65–76)		—	—		94/129	73 (65–80)	
Presence of stunting (height for age below 2 SD)**									
Yes	70/96	76 (64–85)	0.4	—	—	—	42/60	70 (57–80)	0.3
No	111/161	69 (61–77)		—	—		69/88	78 (69–86)	

95% CIs are Wilson 95% confidence intervals of percentage.

*Rao–Scott chi-squared test. For type 2 seroprevalence, children aged 9–12 years were aggregated to allow Rao–Scott *p-*value calculation.

**Wasting or stunting was considered present when weight for height or height for age, respectively, was more than 2 SD below the mean of a standard international reference population recommended by the World Health Organization [15]. Nutritional assessment was available only for a subset of children 1 to 4 years of age.

bOPV, bivalent oral poliovirus vaccine; OPV, oral poliovirus vaccine.

We did not observe higher seroprevalence for types 1 and 3 among younger children who had received more than 3 bOPV doses in the recent campaigns compared with those who had received fewer doses (Table 3). As mentioned above, older children, who had not received bOPV during the campaigns, had similar seropositivity and antibody titers as younger children. Receipt of any OPV in Myanmar was associated with higher type 2 seroprevalence among younger children in the makeshift settlements, as 79% (95% CI 73%–84%) of children who had received OPV in Myanmar were seropositive compared with 60% (95% CI 48%–71%) of children who had not received OPV in Myanmar (*p =* 0.003).

We did not find lower polio immunity among children 1–4 years of age who had signs of acute or chronic malnutrition (Table 3). Finally, there were no seroprevalence differences by sex, and seronegative children were distributed throughout the camp/settlements, without clustering in a specific area.

## Discussion

This cross-sectional assessment among Rohingya children in refugee camps and settlements in Cox’s Bazar found acceptable immunity for type 1 poliovirus among children 1 to 6 years of age after receiving >3 bOPV doses in campaigns conducted in the camps/settlements (85% seropositive in makeshift settlements and 90% in Nayapara). Type 1 seroprevalence was above 90% in children aged 7–14 years, who did not receive vaccine during the campaigns, indicating prior exposure to several OPV doses. Type 2 seroprevalence showed a significant gap in children below 2 years of age, who were born after the global switch from tOPV to bOPV use in routine immunization and campaigns, but was >90% among older children. Finally, type 3 seroprevalence was suboptimal in both age groups and locations (below 75%).

The combination of serological assessment and coverage survey provided information on immunity gaps in specific groups that guided recommendations to the agencies that conducted the campaigns for future immunization activities. First, we were able to confirm that children 7–14 years of age, for whom immunization history was unreliable and who had not received any bOPV in campaigns after arrival to the camps/settlements, had adequate levels of immunity against all serotypes from vaccination in Myanmar. We recommended that future polio vaccination campaigns did not need to target this age group.

Second, our data suggested that children aged below 2 years in makeshift settlements likely remained under-immunized after the campaigns and should receive additional vaccination opportunities. Seroprevalence for type 1 among children aged 1–2 years was about 81% compared to 88% among children aged 3–4 years and 5–6 years. The coverage survey suggested that polio immunity would be even worse among children below 1 year of age. Only 45% of children aged 6–12 months had received >3 bOPV doses in the campaigns, compared with 74%–92% of children aged 1–6 years. Third, the large immunity gap for type 2 poliovirus observed in children aged 1–2 years suggests that many of the children born after the April 2016 tOPV-to-bOPV global switch had not received at least 1 dose of IPV through routine immunization as recommended by WHO and country guidelines [5–7]. This observation agrees with low IPV coverage reported during 2016–2017 in both countries because of the global shortage. IPV coverage in routine immunization was 11% in 2016 and 17% in 2017 in Bangladesh, and 72% in 2016 and 12% in 2017 in Myanmar [4,18,19]. Because of the low seroprevalence observed in younger children, we recommended providing catch-up doses of bOPV and at least 1 dose of IPV to children below 2 years of age, through routine immunization clinics. Addition of bOPV in future campaigns with other antigens targeting children below 5 years of age was also recommended.

Seroprevalence to types 1 and 3 among children below 7 years of age, who had received a median of 4–5 doses of bOPV recently and had been previously exposed to tOPV or bOPV, was below expectations based upon immunogenicity observed with bOPV in clinical trials. A study in India found 86% and 74% seroconversion to types 1 and 3, respectively, after 2 bOPV doses in naïve children, and a study in Bangladesh showed >95% seroconversion to both serotypes after 3 doses [20,21]. Our results suggest that bOPV delivered in campaigns may have lower immunogenicity in certain settings, and this finding will need to be confirmed with new field studies with bOPV. Studies with tOPV have shown conflicting results: Some serosurveys found higher type-specific seroprevalence with tOPV delivered exclusively during campaigns than the same number of doses administered through routine immunization [22], whereas other surveys found lower immunogenicity for doses delivered through campaigns in certain populations [23,24]. Factors known to interfere with the response to tOPV and bOPV, such as diarrhea and malnutrition [25–27], were prevalent in our population [1,12] and may have been partially responsible for the apparent lower effectiveness of bOPV observed. Unfortunately, because of our limitations in accurately calculating the type and number of OPV doses received by each child and the small sample size of subgroups, we were unable to assess the per-dose immunogenicity of bOPV or the potential effects of factors such as malnutrition on seroprevalence [23,27]. The general low reliability of polio vaccination histories to estimate population immunity has also become more relevant with the intensification of immunization campaigns in areas considered at high risk for polio outbreaks, and with the use of different serotype presentations of polio vaccines [28–31].

Polio immunity assessments using serosurveys are necessary to improve the reliability of coverage surveys but may provide insufficient information in populations at high risk. For serum-based serosurveys, skilled health staff need to collect samples by venipuncture, plus staff and equipment that are only available in certain health facilities are necessary to process and store the samples in cold chain. As a result, serosurveys are often conducted among populations visiting or near healthcare facilities, which limits our ability to capture communities whose difficult access to healthcare also makes them more likely to have been missed for immunization [28,32,33].

Compared with serum samples, DBS specimens require additional manipulation and processing in the poliovirus testing laboratory, but have significant advantages for field collection. DBS specimens are easy to obtain from children and adults and do not require collection by skilled health staff. In addition, they are easier to collect and transport than venous blood specimens in remote areas and low-resource settings, because they do not require immediate centrifugation or cold chain [28,32]. DBS-based serosurveys can also be coordinated with other public health activities. We combined our DBS-based serological assessment of several infectious diseases with a nutritional assessment that required blood collection by finger prick for diagnosis of anemia [13]; other groups have conducted DBS serosurveys nested within Demographic and Health Surveys [23,34]. Although combining surveys imposed some restrictions on our choice of sample size and number of vaccine-related questions to include in the questionnaire, savings in the time and resources required for survey implementation compensated for this drawback.

This study has several limitations. The sample was selected to be representative of the Rohingya population settled in Cox’s Bazar, and the results are not generalizable to other populations. Estimation of vaccine doses was mostly based upon parent/caregiver recall of children participating in each vaccination campaign and study assumptions about children receiving bOPV during campaigns, based on the antigens delivered in that campaign and estimated child’s age. Doses received during the September 2017 campaign may have been overestimated because we did not exclude children aged >4 years who might have received measles vaccine only. The selected sample size was not sufficient to assess differences among subpopulations by age, geographical area, or risk factors. We did not conduct multivariable analysis because only age appeared to have some effect on seroprevalence in univariate analysis.

In conclusion, serological testing for polio antibodies in DBS specimens in conjunction with a coverage survey provided a robust assessment of the impact of vaccination activities conducted in Cox’s Bazar, identified children that remained at risk for poliovirus transmission, and informed future vaccination activities. The use of DBS-based serosurveys enhances our ability to guide polio activities in hard-to-access communities at high risk for emergence of VDPV. However, their use must be carefully prioritized, because testing for polio antibodies in DBS specimens is resource intensive and currently can be conducted in only a small number of facilities worldwide [35].

## Supporting information

S1 Data Database and variable dictionary. (XLSX)

S1 Protocol Assessment of vaccination coverage and vaccine-preventable disease serology and exposure to select parasitic diseases among forcibly displaced Rohingyas in Cox’s Bazar, Bangladesh, April–May 2018. (PDF)

S1 STROBE Checklist (PDF)

S1 Vaccination Questionnaire Parent/caregiver vaccination questions, Cox’s Bazar, Bangladesh, April–May 2018. (PDF)

## Abbreviations

bOPV: bivalent oral poliovirus vaccine
cVDPV: circulating vaccine-derived poliovirus
DBS: dried blood spot
IPV: inactivated poliovirus vaccine
OPV: oral poliovirus vaccine
tOPV: trivalent oral poliovirus vaccine
