# Supplementary material for: Assessment of immunity to polio among Rohingya children in Cox’s Bazar, Bangladesh, 2018: A cross-sectional survey
Source: PLoS Med. 2020 Mar 31;17(3):e1003070. doi: 10.1371/journal.pmed.1003070 (PMC7108688; doi:10.1371/journal.pmed.1003070)
Supplement: S1 Vaccination Questionnaire — (PDF) [file pmed.1003070.s004.pdf]

Parent / Infant Caregiver Vaccination Questions
শিশুর পিতামাতা / পরিচর্যাকারির টিকাদান সংক্রান্ত প্রশ্নাবলি
Rohinyga population, Cox's Bazar, Bangladesh
রোহিঙ্গা জনগোষ্ঠি, কক্সবাজার, বাংলাদেশ

000. General Information সাধারণ তথ্য

ID	QUESTION<br>প্রশ্ন	RESPONSE<br>উত্তর	GO TO<br>যান
001	Team number<br>টীম নম্বর	_ _	
002	Name of enumerator/interviewer<br>সাক্ষাতকার গ্রহনকারীর/ তথ্য সংগ্রহকারীর নাম		
003	Date of interview<br>ইন্টারভিউ/ সাক্ষাতের তারিখ	___ ___ / ___ ___ / 2018<br>DD দিন MM মাস/২০১৮	
004	Survey area<br>জরীপ এলাকা	☐1. Kutupalong Registered Camp কতুপালং নি□ন্ধিত ক্যাম্প<br>☐2. Nayapara Registered Camp নয়াপাড়া নি□ন্ধিত ক্যাম্প<br>☐3. Makeshift / Informal Camps অস্থায়ী/অনি□ন্ধিত ক্যাম্প	
005	Block<br>ব্লক		
006	Cluster<br>ক্লাস্টার		
007	Household number<br>খানা নম্বর		
008	Household serial number<br>খানা সিরিয়াল নম্বর		
009	GPS coordinates<br>জিপিএস কোওর্ডিনেট		

Hello. My name is________, and I am working with the Ministry of Health and Family Welfare and partners to learn more about diseases that are affecting the Rohingya community and how they can be prevented by vaccination. What we find during this assessment can help us with future prevention activities, like vaccination campaigns.

We have selected your house randomly and would like to ask you and 1–2 of your children to participate in the assessment. For the selected children in the household (aged 6 months to less than 15 years), we will ask some questions about vaccination, and we may take a few small drops of blood (between 3 and 5 drops). This information will help the Ministry of Health and Family Welfare better understand if you and your community are protected against diseases such as measles, diphtheria, and malaria, and it will help to better plan for health, vaccination, and other services for your community in the future.

Whether you choose to participate in this study is entirely up to you. Participation is completely voluntary. You can also choose to stop your/your child's participation at any time or withdraw from the study.

The information you share with us will be kept confidential. The questions and fingerprick take about 10–15 minutes per child. Do you have any questions? May I begin?

আস্‌সালামুআলাইকুম/ আদাব। আমি ---------------------- । আমি বর্তমানে কক্সবাজার জেলায় অবস্থানকারী বলপূর্বক বাস্তুচ্যুত মিয়ানমার এর নাগরিকদের মধ্যে টিকাদান ও টিকা প্রদানের মাধ্যমে প্রতিরোধযোগ্য রোগ সম্পর্কিত গবেষণা করছি।

গবেষণার সারসংক্ষেপ ঃ
টিকাদান কর্মসূচী বাংলাদেশের একটি সফলতম কার্যক্রম। কক্সবাজার জেলায় অবস্থানকারী বলপূর্বক বাস্তুচ্যুত মিয়ানমার এর নাগরিকদের ৬ মাস হতে ১৫ বছর এর কম বয়সী শিশুদের কয়েকটি টিকাদান অভিযানে সময় টিকা প্রদানের কর্মসূচির মাধ্যমে প্রতিরোধ যোগ্য রোগসমুহ প্রতিরোধে টিকা প্রদান করা হচ্ছে। আমরা এ গবেষণার মাধ্যমে টিকা প্রদান পরবর্তী শিশুদের অর্জিত রোগ প্রতিরোধ ক্ষমতা এবং টিকাদান অভিযানে অংশ না নেয়ার কারণ সম্পর্কে জানতে আগ্রহী।

এ গবেষণা কার্যক্রমে কেন আপনাকে নির্বাচিত করা হলো?
আমাদের গবেষণা কার্যক্রম সেই সকল শিশুদের উপর পরিচালিত হচ্ছে, যাদের বয়স ৬ মাস থেকে ১৫ বছরের মধ্যে, বলপূর্বক বাস্তুচ্যুত মিয়ানমার এর নাগরিক এবং কক্সবাজার জেলায় অবস্থানকারী । আপনি এ রকমের একজন শিশুর অভিভাবক হওয়া তে আপনাকে সাক্ষাৎকারের জন্য নির্বাচিত করা হয়েছে।

অংশ গ্রহনের ধরণঃ
আপনি যদি আমাদের গবেষণা কার্যক্রমে অংশগ্রহনের সম্মতি প্রদান করেন, তাহলে আমরা আপনার একটি সাক্ষাৎকার গ্রহণ করব। সাক্ষাৎকারটি দিতে আপনার ১৫ মিনিটের মতো সময় লাগবে। সাক্ষাৎকারে আমরা আপনার আর্থ-সামাজিক অবস্থা, শিশুর টিকা প্রদান সম্পর্কিত তথ্য এবং শিশুর টিকা প্রদানের সমস্যা সম্পর্কিত কিছু প্রশ্ন করব। এ ছাড়া শিশুর নিকট হকে ৩-৪ ফোঁটা রক্ত সংগ্রহকরব।
সাক্ষাৎকার চলাকালে যে কোনো সময়ে আপনি ইচ্ছা করলে সাক্ষাৎকার প্রদান থেকে নিজেকে বিরত রাখতে পারেন।

অংশ গ্রহনের ঝুঁকি এবং প্রাপ্ত সুবিধাসমুহঃ
আমাদের গবেষণা কার্যক্রমে অংশ গ্রহনে আপনার / আপনার শিশুর অথবা আপনার পরিবারের কোন ঝুঁকি নেই। আমাদের গবেষণা কার্যক্রমে অংশগ্রহন করে আপনি কোনো প্রকার ব্যক্তিগত অথবা আর্থিক সুবিধা পাবেন না। শিশুর রক্ত সংগ্রহের সময় সামান্য ব্যথা লাগতে পারে, যা সাময়িক, এ সংক্রান্ত অন্য জটিলতা হলে আমরা প্রয়োজনীয় ব্যবস্থা নিব।

ব্যক্তিগত পরিচিতি ও প্রদত্ত তথ্যের গোপনীয়তাঃ
আমরা আপনার / আপনার শিশুর ব্যক্তিগত পরিচিতি ও প্রদত্ত তথ্যের গোপনীয়তা রক্ষা করার প্রতিশ্রুতি দিচ্ছি। আপনি ইচ্ছা করলে তথ্য প্রদান থেকে নিজেকে বিরত রাখতে পারেন, সে ব্যাপারে আপনার সম্পূর্ণ স্বাধীনতা আছে।
আমরা নিশ্চয়তা দিচ্ছি যে, তথ্য-উপাত্ত বিশ্লেষণের সময় এবং রিপোর্ট প্রকাশের সময় আপনার / আপনার শিশুর অথবা আপনার পরিবারের নাম ও ঠিকানা সম্পূর্ণ রূপে গোপন রাখা হবে। সকল সাক্ষাৎকার ও তথ্য-উপাত্ত আমাদের অফিসে তালাবদ্ধ থাকবে।

তথ্য-উপাত্তের ভবিষ্যৎ ব্যবহার ঃ
আমরা নিশ্চয়তা দিচ্ছি যে, ভবিষ্যতে যে কোনো সময়ে আপনার প্রদত্ত তথ্য-উপাত্ত পুনঃব্যবহার করা হলে আপনার পরিচিতি অবশ্যই গোপন রাখা হবে।

তথ্য প্রদানে অসম্মতি জ্ঞাপনের স্বাধীনতা ঃ
এই গবেষণা কার্যক্রমে আপনার / আপনার শিশুর অংশগ্রহন নিজের ইচ্ছার উপর নির্ভরশীল। আপনি ইচ্ছা করলে তথ্য প্রদান থেকে নিজেকে বিরত রাখতে পারেন, সে ব্যাপারে আপনার সম্পূর্ণ স্বাধীনতা আছে। তথ্য প্রদান থেকে বিরত থাকলে, আপনি কোনো রকম আইনী জটিলতায় পড়বেন না।

ক্ষতিপূরণ প্রাপ্তির অধিকার ঃ
এই গবেষণা কার্যক্রমে আপনার অংশগ্রহন করে আপনি কোনোরকম ব্যক্তিগত অথবা আর্থিক ক্ষতির সম্মুখীন হবেন না বিধায়, এই গবেষণাকার্যক্রম থেকে, কোনো রকম ব্যক্তিগত অথবা আর্থিক ক্ষতিপূরণ দাবী করতে পারবেন না। তবে শিশুর রক্ত সংগ্রহের সংক্রান্ত কোনো জটিলতা হলে আমরা প্রয়োজনীয় ব্যবস্থা নিব।

এই গবেষণা কার্যক্রম সম্পর্কে আরও কিছু জিজ্ঞাসা থাকলে আইইডিসিআর বরাবরে যোগাযোগ করবেন।আপনি যদি উপরোক্ত তথ্যাবলী সম্পূর্ণরূপে বুঝে, আমাদের গবেষণা কার্যক্রমে অংশগ্রহনের সম্মত থাকেন তবে আপনাকে স্বাক্ষর/ টিপসহি প্রদান করতে হবে। আমরা এই সম্মতিপত্রের এক কপি আপনাকে দিয়ে যাবো।

গবেষণা কার্যক্রমে অংশগ্রহনের জন্য আপনাকে ধন্যবাদ।

আমি কি সাক্ষাতকারটি শুরু করতে পারি?

IF CONSENT REFUSED: Please ensure that Team Leader has explained clearly the objectives of the survey. If the head of household/respondent still refuses, **go to end of questionnaire**.

সম্মতি না প্রদান করলে- গবেষণার উদ্দেশ্য সম্পর্কে পরিস্কারভাবে জানাতে হবে। এর পরও খানা প্রধান / উত্তরদাতা সম্মতি প্রদান না করলে- প্রশ্ন পত্রের শেষে যান।

First, Can you share with me any documents and cards you have received related to the vaccinations and health facility or hospital visits for your children? *Interviewer should show what cards he/she is interested in seeing.*

প্রথমত, আপনার সন্তানদের টিকাদান, স্বাস্থ্য ব্যাবস্থা বা হাসপাতালে ভর্তি সম্পর্কিত কোনও দলিল এবং কার্ড থাকলে আমাকে দেখতে দিন। সাক্ষাতকারগ্রহনকারির দেখানো উচিত কোন কার্ডগুলি তিনি দেখতে আগ্রহী।.

100. Vaccination of Child Aged 6 months to 6 years
শিশুকে টিকাদান- ৬ মাস থেকে ৭ বছর বয়স পর্যন্ত

I would now like to ask you about your child's vaccination history.
আমি এখন আপনাকে আপনার সন্তানের টিকা সম্পর্কে কিছু জিজ্ঞাসা করতে চাই।

Ask child's name and substitute "this child" with the name of the child in this section.
সন্তানের নাম জিজ্ঞাসা করুন এবং এই বিভাগে "এই শিশুর" স্থলে সন্তানের নাম ব্যবহার করুন।

ID	QUESTION<br>প্রশ্ন	RESPONSE<br>উত্তর	GO TO<br>যান
101	Child #<br>শিশুর নম্বর	___ ___ ___ ___ ___ ___	
102	Child age<br>শিশুটির □য়স	______ months / years *(circle)*<br>মাস / □ছর (□ৃত্ত আঁকুন)<br><br>☐88. Refused to answer উত্তর দিতে রাজি না	
103	Is the child present?<br>*আপনার শিশুটি এখানে উপস্থিত ?*	☐1. Yes হ্যাঁ<br>☐2. No না	
104	What is your relationship with the selected child?<br><br>নির্□াচিত শিশুর সঙ্গে আপনার সম্পর্ক কি?<br><br>*The respondent may consult with his/her spouse, children, neighbors, etc.*<br><br>উত্তরদাতআ তার স্বামী/ স্ত্রী, শিশু, প্রতি□েশী, ইত্যাদির সঙ্গে পরামর্শ করতে পার□েন	☐1. Mother মা<br>☐2. Father □া□া<br>☐3. Grandmother দাদী/ নানী<br>☐4. Grandfather দাদা/নানা<br>☐5. Aunt খালা/ ফুপু/ চাচী/কাকী/মামী<br>☐6. Uncle খালু/ফুপা/ চাচা/কাকা/মামা<br>☐7. Sister □োন<br>☐8. Brother ভাই<br>☐9. Other relative অন্যান্য অন আত্মীয়<br>☐10. Other অন্যান্য<br>☐88. Refused to answer<br>উত্তর দিতে অস□ীকৃতি জানানো	
105	Did this child arrive in Bangladesh together with the family?<br><br>শিশুটি কি পরি□ার সহ □াংলাদেশে এসেছিল ?	☐1. Yes হ্যাঁ --><br>☐2. No না<br>☐88. Refused to answer --------------------------><br>উত্তর দিতে অস□ীকৃতি জানানো<br>☐99. Don't know জানি না -------------------------->	Q107<br><br>Q107<br><br>Q107
106	If no, (the child arrived earlier or later), when did the child arrive in Bangladesh?<br><br>উত্তর না হলে (শিশুটি আগে/পরে আসলে) শিশুটি ক□ে □াংলাদেশে এসেছিল ?<br><br>*Dates can be calculated based on Kurbani Eid (01 Sep 2017). Estimate the number of lunar months after Eid the child arrived.*<br><br>*কুর□ানির ঈদ (১ সেপ্টেম্বর, ২০১৭) থেকে তারিখ গণনা করা যেতে পারে।*	___ ___ lunar cycles after EID<br><br>ঈদের _____ চন্দ্রমাস পর<br><br>☐88. Refused to answer উত্তর দিতে অস্বীকার<br>☐99. Don't know জানি না	

	ঈদের পর থেকে চন্দ্রমাস গণনা করুন।		
107	Who takes the decision to vaccinate or not vaccinate this child?<br><br>আপনার শিশুর টিকা দেয়া / না দেয়া সিদ্ধান্ত কে নেয় ?<br><br>*Do not reach choices. Mark all that are mentioned.*<br><br>পছন্দগুলো বলবনে না। □াদের নাম বলা হয় সবগুলো লিপিবদ্ধ করুন ন।	☐1. Mother মা<br>☐2. Father বাবা<br>☐3. Sister বোন<br>☐4. Brother ভাই<br>☐5. Other female relative<br>অন্য কোন মহিলা আত্মীয়<br>☐6. Other male relative<br>অন্য কোন পুরুষ আত্মীয়<br>☐7. Other অন্যান্য<br>☐8. The child by himself or herself শিশু নিজেই<br>☐9. No one কেউ না<br>☐88. Refused to answer উত্তর দিতে অস্বীকার<br>☐99. Don't know জানি না	

If card is available, use date on vaccination card to answer questions. If no date is written on the card(s) or if card is missing, ask corresponding questions. For these questions, it may be helpful to use the event of Kurbani Eid, 01 Sep 2017, (or their arrival date if close to Kurbani Eid) to associate the first 2 campaigns (MR 1st round and OCV 1st round). As a reference, children under 2 years of age should have received injections in the thigh. Children 2 years and older should have received injections in the arm.

টিকাদান কার্ডটি থাকলে, টিকার তারিখ কার্ডে উল্লিখিত তারিখ লিখুন। টিকাদান কার্ডে তারিখ না থাকলে অথ□া কার্ডটি হারিয়ে গেলে, নিচের টিকার তারিখ সম্পর্কিত প্রশ্নগুলো করুন।

এই প্রশ্নগুলি প্রথম দুটো টিকাদান ক্যাম্পেইন (এমআর ১ম রাউন্ড এ□ং ওসিভি ১ম রাউন্ড) এর সাথে সং□ুক্ত করার জন্য কোর□ানি ঈদ ০১ সেপ্টেম্বর ২০১৭, (অথ□া তাদের আগমনের তারিখ □দি কোর□ানি ঈদের কাছাকাছি হয়) □্□হার করা □েতে পারে।

একটি রেফারেন্স হিসা□ে □লা □ায়, ২ □ছরের কম □য়সী শিশুদের ঊরুতে এ□ং ২ □ছর এ□ং তার উপরের □য়সের শিশুদের □াহুতে ইনজেকশন পাওয়া উচিত।

	How many times did this child take diphtheria vaccine?<br>এই শিশুটি কতবার ডিপথেরিয়ার টিকা নিয়েছিল?<br><br>*This question is asked to guide the next few questions and will not be recorded.*<br><br>পরবর্তী কয়েকটি প্রশ্নের দিক নির্দেশনার এই প্রশ্নটি করা হবে কিন্তু রেকর্ড করা হবে না।		
108	About a month ago, there was a diphtheria vaccination campaign that included **one diphtheria injection** and **oral polio drops**. Did this child receive an injection and oral drops? This child might have had **pain on touch** and maybe slight fever, which is normal. You may have received a **vaccination card** as well.<br><br>*(10–29 March 2018: Penta+OPV, 3rd round for 6weeks–<7years)*	☐ 1. Yes, confirmed by date in vaccination card ---><br>হ্যাঁ, টিকা কার্ডের তারিখ অনুযায়ী নিশ্চিত<br><br>☐ 2. Yes, by recall -----------------------------------><br>হ্যাঁ, সাক্ষাতকারী প্রদানকারী মনে করে □ললেন<br><br>☐3. No না<br><br>☐88. Refused to answer উত্তর দিতে রাজি না ------><br><br>☐99. Don't know জানি না --------------------------->	Q110<br><br>Q110<br><br><br><br>Q111<br><br>Q111

	প্রায় এক মাস আগে একটি ডিপথেরিয়া টিকাদান অভি□ান হয়েছিল, □খানে একটি ডিপথেরিয়া ইনজেকশন ও পোলিও ড্রপ মুখে খাওয়ানো হয়েছিল।<br>আপনার শিশুটি কি সেখানে একটি ডিপথেরিয়া ইনজেকশন ও একটি পোলিও ড্রপ মুখে খেয়েছিল্ ?<br>টিকাদান পর□র্তী সময়ে, সামান্য □্যাথা অথ□া সামান্য জ্বর হতে পারে, □া স্বাভা□িক। আপনি সেখানে একটি টিকাদান কার্ডও পেতে পারেন (মার্চ, ২০১৮: পেন্টা + ওপিভি ৩য় রাউন্ড- ৬ সপ্তাহ- অনুর্ধ্ব ৭ □ছর □য়সী শিশুর জন্য)		
109	What are the reasons why the child did not get vaccinated?<br>শিশুটির টিকা না পাওয়ার কারণগুলো কি ?<br><br>*Instructions:*<br>*The respondent should be prompted: 'Are there any other reasons?' until they have mentioned all reasons.*<br><br>নির্দেশা□লী:<br>উত্তরদাতাকে অনুরোধ করা উচিত: *' আরও কোন কারণ আছে কিনা'* ?<br><br>*Do not read choices. Mark all that are mentioned.*<br><br>পছন্দগুলি পড়□েন না । সেগুলো উল্লেখ কর□ে সেস□ চিহ্নিত করুন।	☐1. Caregiver was unaware of the vaccination campaign<br>পরিচর্□াকারি টিকাদান ক্যাম্পেইন সম্পর্কে সচেতন ছিলনা<br>☐2. Caregiver thinks that child received all required vaccines<br>পরিচর্□াকারি মনে করে □ে শিশুটি স□ প্রয়োজনীয় টিকা গ্রহন করেছে<br>☐3. Caregiver does not think vaccines are necessary<br>পরিচর্□াকারি চান না □ে শিশুটি টিকা গ্রহন করুক<br>☐4. Caregiver is fearful of vaccines or fearful of multiple injections<br>পরিচর্□াকারী অনেকগুলো ইনজেকশন এক সঙ্গে দিতে হ□ে, তাই ভয় পেয়েছিলেন<br>☐5. Caregiver or child is fearful about not going to heaven or being converted to Christianity<br>পরিচর্□াকারী জান্নাতে না □েতে পারা অথ□া খ্রীস্ট ধর্মে রূপান্তরিত হওয়ার ভয় পেয়েছেন<br>☐6. Female caregiver or child not comfortable leaving the house<br>মহিলা পরিচর্□াকারী অথ□া শিশুটির পক্ষে ঘরটি ছেড়ে □াওয়া সম্ভ□পর ছিল না<br><br>☐7. Female caregiver or child not comfortable taking vaccine from a male vaccinator<br>মহিলা পরিচর্□াকারী অথ□া শিশুটির নিকট পুরুষ টিকাদানকারী গ্রহন□োগ্য ছিল না<br>☐8. The child got fever/swelling after last vaccination<br>শিশুটির শেষ টিকা গ্রহনের পর জ্বর / টিকার স্থানের ফুলে গিয়েছিল<br><br>☐9. No one was available to take the child to vaccination.<br>শিশুকে টিকা দিতে নিয়ে □াওয়ার জন্য কেউ ছিলনা	Q111

		☐10. Child was not available (e.g., not at home, sick) at time of vaccination<br>শিশুটি □াসায় ছিল না / অসুস্থ ছিল<br>☐11. Child was afraid of needles/pain<br>শিশুটি ইনজেকশন/ □্যাথার ভয় পেয়েছিল<br>☐12. Father or head of household does not allow<br>শিশুর পিতা / পরি□ার প্রধান অনুমতি দেয়নি<br>☐13. Vaccinator treated caregiver or child poorly<br>টিকাদান কারী শিশু/ পরিচর্□াকারীর সাথে ভালো □্য□হার করেননি<br>☐14. Other অন্যান্য<br>☐88. Refused to answer উত্তর দিতে অস্বীকার<br>☐99. Don't know জানি না	
110	Who took the child to get vaccinated?<br><br>টিকা দিতে কে শিশুকে নিয়ে গিয়েছিল?<br><br>*Do not reach choices. Mark only one.*<br><br>পছন্দগুলো পড়ি□েন না, শুধুমাত্র একটিতে টিক চিহ্ন দিন।	☐1. Mother মা<br>☐2. Father □া□া<br>☐3. Sister □োন<br>☐4. Brother ভাই<br>☐5. Other female relative<br>অন্য কোন মহিলা আত্মীয়<br>☐6. Other male relative অন্য কোন পুরুষ আত্মীয়<br>☐7. Neighbor/friend প্রতি□েশী / □ন্ধু<br>☐8. Social mobilizer সমাজসে□ী<br>☐9. Vaccinated at school<br>স্কুলে টিকা দেয়া হয়েছিল<br>☐10. Vaccinated at madrassa<br>মাদ্রাসায় টিকা দেয়া হয়েছিল<br>☐11. Child went by him/her self শিশু নিজে গিয়েছে<br>☐12. Other অন্যান্য ---------------------<br>☐88. Refused to answer উত্তর দিতে অস্বীকার<br>☐99. Don't know জানি না	
111	Before the last campaign, there was another diphtheria vaccination campaign that included **<u>one diphtheria injection</u>** and **<u>oral polio drops</u>**. Did this child receive an injection and oral drops? This child might have had **<u>pain on touch</u>** and maybe slight fever, which is normal. You may have received a **<u>vaccination card</u>** as well.<br><br>গত টিকাদান অভিযান এর আগে, আরও একটি ডিপথেরিয়া টিকাদান অভিযান হয়েছিল, যেখানে একটি ডিপথেরিয়া ইনজেকশন ও পোলিও ড্রপ মুখে খাওয়ানো হয়েছিল।<br>আপনার শিশুটি কি সেখানে একটি ডিপথেরিয়া ইনজেকশন ও একটি পোলিও ড্রপ মুখে খেয়েছিল্ ?<br>টিকাদান পর□র্তী সময়ে, সামান্য □্যাথা অথ□া সামান্য জ্বর হতে পারে, যা	☐1. Yes, confirmed by date in vaccination card<br>হ্যাঁ, টিকা কার্ডের তারিখ অনুযায়ী নিশ্চিত<br>☐2. Yes, by recall হ্যাঁ, স্মরন দ্বারা<br>☐3. No না<br>☐88. Refused to answer উত্তর দিতে রাজি না<br>☐99. Don't know জানি না	

	স্বাভাবিক। আপনি সেখানে একটি টিকাদান কার্ডও পেতে পারেন<br><br>*(27 January–10 February 2018: Penta+OPV, 2nd round for 6weeks–<7years)*<br><br>(২৭ জানুয়ারী থেকে ১০ ফেব্রুয়ারী, ২০১৮: পেন্টা + ওপিভি ২য় রাউন্ড- ৬ সপ্তাহ- অনুর্ধ্ব ৭ বছর বয়সী শিশুর জন্য)		
112	And before that campaign, there was another **diphtheria** vaccination campaign that included **two injections, one in each thigh or arm,** and **oral polio drops**. Did this child receive the two injections and oral drops?<br><br>This child might have had **pain on touch** and maybe slight fever, which is normal. You may have received a **vaccination card** as well.<br><br>*(12–31 December 2017: Penta+PCV+OPV, 1st round for 6weeks–<7years)*<br><br>এবং সেই ক্যাম্পেইনের আগে, আরও একটি ডিপথেরিয়া টিকাদান অভিযান হয়েছিল, যেখানে প্রত্যেক উরু অথবা বাহুতে দুইটি ইনজেকশন ও পোলিও ড্রপ মুখে খাওয়ানো হয়েছিল। আপনার শিশুটি কি সেখানে দুইটি ইনজেকশন ও একটি পোলিও ড্রপ মুখে খেয়েছিল্ ?<br><br>টিকাদান পরবর্তী সময়ে, সামান্য ব্যাথা অথবা সামান্য জ্বর হতে পারে, যা স্বাভাবিক। আপনি সেখানে একটি টিকাদান কার্ডও পেতে পারেন<br><br>(১২-৩১ ডিসেম্বর, ২০১৭: পেন্টা +পিসিভি+ ওপিভি ১ম রাউন্ড- ৬ সপ্তাহ - অনুর্ধ্ব ৭ বছর বয়সী শিশুর জন্য)	☐1. Yes, confirmed by date in vaccination card হ্যাঁ, টিকা কার্ডের তারিখ অনুযায়ী নিশ্চিত<br>☐2. Yes, by recall হ্যাঁ, স্মরন দ্বারা<br>☐3. No না<br>☐88. Refused to answer উত্তর দিতে রাজি না<br>☐99. Don't know জানি না	
	How many times did this child take measles vaccine?<br><br>এই শিশুটি কতবার হামের টিকা নিয়েছে?		

	This question is asked to guide the next few questions and will not be recorded.<br><br>এই প্রশ্নটি পরবর্তী কয়েকটি প্রশ্নের দিক নির্দেশনার জন্য বলা হয়েছে এবং এটি রেকর্ড করা হবে না।		
113	Before the diphtheria vaccination campaign, there was a vaccination campaign that included **one injection for measles and NO oral drops**. Did this child receive the injection? You may have received a **vaccination card** as well.<br><br>ডিপথেরিয়া টিকাদান অভিযান এর আগে আরও একটি অভিযান হয়েছিল, যেখানে একটি হামের টিকার ইনজেকশন দেয়া হয়েছিল, কোনো পোলিও ড্রপ মুখে খাওয়ানো হযনি। আপনার শিশুটি কি টিকা পেয়েছিল ? আপনি সেখানে একটি টিকাদান কার্ডও পেতে পারেন ।<br><br>*(18 November–05 December 2017: MR, 2nd round for 6months–<15years)*<br><br>(১৮ নভেম্বর থেকে ৫ ডিসেম্বর, ২০১৭ এম আর ২য় রাউন্ড- ৬ মাস- অনুর্ধ্ব ১৫ বছর বয়সী শিশুর জন্য)	☐1. Yes, confirmed by date in vaccination card হ্যাঁ, টিকা কার্ডের তারিখ অনুযায়ী নিশ্চিত<br>☐2. Yes, by recall হ্যাঁ, স্মরন দ্বারা<br>☐3. No না<br>☐88. Refused to answer উত্তর দিতে রাজি না<br>☐99. Don't know জানি না	
114	There was another measles vaccination campaign a few weeks after Kurbani Eid that included cutting of the **Vitamin A red pill** for young children, **measles injection** and **oral polio drops**. Did this child receive the injection and oral drops? You may have received a **vaccination card** as well.<br><br>কুরবানির ঈদেব কয়েক সপ্তাহ পরে আরও একটি হামের টিকাদান অভিযান হয়েছিল, যেখানে একটি ভিটামিন এ এর লাল পিল, একটি হামের টিকার ইনজেকশন দেয়া হয়েছিল ও পোলিও ড্রপ মুখে খাওয়ানো হয়েছিল ।<br>আপনার শিশুটি কি টিকাগুলো পেয়েছিল ?<br><br>আপনি সেখানে একটি টিকাদান কার্ডও পেতে পারেন ।	☐1. Yes, confirmed by date in vaccination card হ্যাঁ, টিকা কার্ডের তারিখ অনুযায়ী নিশ্চিত<br>☐2. Yes, by recall হ্যাঁ, স্মরন দ্বারা<br>☐3. No না<br>☐88. Refused to answer উত্তর দিতে রাজি না<br>☐99. Don't know জানি না	

	(16 September–03 October 2017:<br>OPV only for 0–<6months<br>OR MR+OPV+Vitamin A, 1st round for 6months–<5years OR MR only for 5years–<15years)<br>(১৬ সেপ্টেম্বর থেকে ০৩ অক্টোবর, ২০১৭:<br>ওপিভি-০ থেকে অনুর্ধ্ব ৬ মাস<br>ভিটামির এ+ এম আর + ওপিভি- ৬ মাস- অনুর্ধ্ব ৫ বছর বয়সী শিশুর জন্য<br>এম আর ১ম রাউন্ড ৫ বছর -অনুর্ধ্ব ১৫ বছর বয়সী শিশুর জন্য)		
	How many times did this child take oral cholera vaccine from a small bottle?<br>আপনার শিশুকে কত বার ছোট একটি বোতল থেকে কলেরা টিকা মুখে খাওয়ানো হয়েছিল ?<br><br>*This question is asked to guide the next few questions and will not be recorded.*<br>এই প্রশ্নটি পরবর্তী কয়েকটি প্রশ্নের দিক নির্দেশনার জন্য বলা হয়েছে এবং এটি রেকর্ড করা হবে না।		
115	After the Vitamin A / measles campaign, there was a vaccination campaign that included **<u>oral cholera vaccine in a small bottle for both adults and children</u>** (from 1 year and above). Did this child receive the oral vaccine? You may have received **<u>soap</u>** as well.<br>ভিটামিন এ ও হামের টিকাদান অভিযান এর পরে একটি অভিযান হয়েছিল, যেখানে একটি ছোট একটি □োতল থেকে কলেরা টিকা □ড় ও শিশুদের (১□ছর ও তদুর্ধ্ব) খাওয়ানো হয়েছিল।<br>আপনার শিশুটি কি কলেরা টিকা পেয়েছিল ?<br>আপনি সেখানে একটি সা□নও পেতে পারেন।<br><br>*(10–18 October 2017:<br>OCV, 1st round for ≥1year)*<br>(১০-১৮ অক্টো□র, ২০১৭: ওসিভি ১ম রাউন্ড- ১□ছর ও তদুর্ধ্ব)	☐1. Yes হ্যাঁ<br>☐2. No না<br>☐88. Refused to answer উত্তর দিতে রাজি না<br>☐99. Don't know জানি না	
116	After the oral cholera vaccine campaign that included soap, there was another	☐1. Yes হ্যাঁ<br>☐2. No না	

	vaccination campaign that included **<u>oral cholera vaccine in a small bottle</u>** but only for children older than 1 but less than 5 years old. Did this child receive the oral vaccine?<br>কলেরা টিকাদান অভিযান, যেখানে একটি সাবান দেয়া হয়েছিল, তার পরে একটি অভিযান হয়েছিল, যেখানে একটি ছোট একটি বোতল থেকে কলেরা টিকা ১-৫বছর বয়সী শিশুদের খাওয়ানো হয়েছিল।<br>আপনার শিশুটিকে কি কলেরা টিকা খাওয়ানো হয়েছিল।<br><br>*(04–09 November 2017: OCV+OPV, 2nd round for 1year–<5years OR OPV only for <1year)*<br>(০৪-০৯ নভেম্বর, ২০১৭: ওসিভি + ওপিভি ২য় রাউন্ড- ১ বছর -৫বছর এবং শুধুমাত্র ওপিভি অনুর্ধ্ব ১ বছর বয়সী শিশু)	☐88. Refused to answer উত্তর দিতে রাজি না<br>☐99. Don't know জানি না	
117	When you and your family entered Bangladesh, did this child take any vaccination at the border, either injections or oral polio drops?<br><br>আপনি এবং আপনার পরিবার যখন বাংলাদেশে এসেছিলেন, তখন সীমান্তে আপনার শিশু কি কোনো ইনজেকশন অথবা মুখে পোলিও টিকা খাওয়ানো হয়েছিলো?	☐1. Yes, confirmed by date in vaccination card হ্যাঁ, টিকা কার্ডের তারিখ অনুযায়ী নিশ্চিত<br>☐2. Yes, by recall হ্যাঁ, স্মরন দ্বারা<br>☐3. No না<br>☐88. Refused to answer উত্তর দিতে রাজি না<br>☐99. Don't know জানি না	
118	Can you share any additional vaccination cards or medical records related to this child?<br>আপনি কি এই শিশুর সাথে সম্পর্কিত কোনও অতিরিক্ত টিকাদান কার্ড বা মেডিকেল রেকর্ড দেখাতে পারেন?<br><br>*If there are any additional vaccination cards that do not correspond to campaign doses, vaccination at the border or vaccination for being a diphtheria contact, indicate which vaccines were received.*<br>যদি কোনো অতিরিক্ত টিকাদান কার্ড থাকে যা ক্যাম্পেইন ডোজ, সীমান্তে টিকা বা ডিপথেরিয়ার কারনে টিকার সাথে সম্পর্কিত নয় তাহলে, কোন টিকা তা নির্দেশ করুন।	☐1. OPV, confirmed by date in vaccination card ওপিভি, টিকা কার্ডের তারিখ অনুযায়ী নিশ্চিত<br>☐2. PCV, confirmed by date in vaccination card পিসিভি টিকা কার্ডের তারিখ দ্বারা নিশ্চিত<br>☐3. Penta, confirmed by date in vaccination card পেন্টা টিকা কার্ডের তারিখ অনুযায়ী নিশ্চিত<br>☐4. Td, confirmed by date in vaccination card টিডি, টিকা কার্ডের তারিখ দ্বারা নিশ্চিত<br>☐5. MR, confirmed by date in vaccination card এম.আর.টিকা কার্ডের তারিখ অনুযায়ী নিশ্চিত<br>☐6. BCG, confirmed by date in vaccination card বিসিজি, টিকাদান কার্ড থেকে নিশ্চিত<br>☐7. IPV, confirmed by date in vaccination card আইপিভি, টিকাদান কার্ড থেকে নিশ্চিত<br>☐8. Other অন্যান্য<br>☐9. No other vaccination card available অন্য কোন টিকা কার্ড পাওয়া যায় না	
119	When this child lived in Myanmar, did this child ever take oral polio drops. Polio is a	☐1. Yes, confirmed by date in vaccination card হ্যাঁ, টিকা কার্ডের তারিখ অনুযায়ী নিশ্চিত	

	vaccine given by dropping liquid into a child’s mouth. Oral polio drops are different from Vitamin A drops, which usually are red or blue pills.<br>যখন এই শিশুটি মায়ানমারে □স□াস করত, তখন এই শিশুটি কি কখনো পোলিও টিকা □া মৌখিক পোলিও ড্রপ গ্রহন করেছে? পোলিও একটি তরল টিকা যা শিশুর মুখের মধ্যে ড্রপ দ্বারা দেওয়া হয়।	☐2. Yes, by recall হ্যাঁ, স্মরন দ্বারা<br>☐3. No না<br>☐88. Refused to answer উত্তর দিতে রাজি না<br>☐99. Don’t know জানি না	
120	When this child lived in Myanmar, did this child ever take any vaccination through injection?<br>যখন এই শিশু মায়ানমারে □স□াস করত, তখন কি এই শিশুটি ইনজেকশন এর মাধ্যমে কোনো টিকা নিয়েছে ?	☐1. Yes, confirmed by date in vaccination card<br>হ্যাঁ, টিকা কার্ডের তারিখ অনুযায়ী নিশ্চিত<br>☐2. Yes, by recall হ্যাঁ, স্মরন দ্বারা<br>☐3. No না<br>☐88. Refused to answer উত্তর দিতে রাজি না<br>☐99. Don’t know জানি না	
121	*Does the child have a BCG scar? Check the child’s upper right and left arms. (Do not ask the caregiver.)*<br><br>এই শিশুটির কি *বিসিজি টিকার চিহ্ন* আছে? শিশুর ডান এবং বাম বাহুর উপরে পরীক্ষা করুন। (পরিচর্যাকারিকে জিজ্ঞাসা করবেন না।)	☐1. Yes হ্যাঁ<br>☐2. No না<br>☐3. Not sure if scar is from BCG<br>নিশ্চিত না স্কারটি বিসিজির কিনা<br>☐99. Child not available শিশুটি উপস্থিত নাই	
122	In the past two weeks, has this child had a fever without rash?<br>বিগত ২ সপ্তাহে কি শিশুর র‍্যাশ ছাড়া জ্বর হয়েছিলো ?	☐1. Yes হ্যাঁ<br>☐2. No না<br>☐88. Refused to answer উত্তর দিতে রাজি না<br>☐99. Don’t know জানি না	
123	After arrival in Bangladesh, has this child had measles?<br>বাংলাদেশে আগমনের পর, শিশুর কি কখনো হাম হয়েছিলো ?	☐1. Yes, confirmed by health facility document<br>হ্যাঁ, স্বাস্থ্য কেন্দ্রের দলিল অনু□ায়ী নিশ্চিত<br>☐2. Yes, verbal report that the child was diagnosed at a clinic<br>হ্যাঁ, কোনো ক্লিনিকে মৌখিকভাবে নিশ্চিত<br>☐3. Yes, verbal report that the child was diagnosed by a local healer<br>হ্যাঁ, কোনো স্থানীয় পল্লী চিকিসক কর্তৃক মৌখিকভাবে নিশ্চিত<br>☐4. Yes, verbal report but did not seek diagnosis<br>হ্যাঁ, মৌখিক রিপোর্ট কিন্তু ওই রোগের জন্য □ায়নি<br>☐5. No না<br>☐88. Refused to answer উত্তর দিতে রাজি না<br>☐99. Don’t know জানি না	
124	After arrival in Bangladesh, has this child had diphtheria?<br><br>বাংলাদেশে আগমনের পর, শিশুর কি কখনো ডিপথেরিয়া হয়েছিলো ?	☐1. Yes, confirmed by health facility document<br>হ্যাঁ, স্বাস্থ্য কেন্দ্রের দলিল অনু□ায়ী নিশ্চিত<br>☐2. Yes, verbal report that the child was diagnosed at a clinic<br>হ্যাঁ, কোনো ক্লিনিকে মৌখিকভাবে নিশ্চিত<br>☐3. Yes, verbal report that the child was diagnosed by a local healer<br>হ্যাঁ, কোনো স্থানীয় পল্লী চিকিসক কর্তৃক মৌখিকভাবে নিশ্চিত<br>☐4. Yes, verbal report but did not seek diagnosis	

		হ্যাঁ, মৌখিক রিপোর্ট কিন্তু ওই রোগের ক্লিনিকে জন্য যায়নি<br>☐5. No না<br>☐88. Refused to answer উত্তর দিতে রাজি না<br>☐99. Don't know জানি না	

200. Vaccination of Child Aged 7years to 14years শিশুকে টিকাদান ৭-১৫ বছর বয়স পর্যন্ত

I would now like to ask you about your child's vaccination history.
আমি এখন আপনার সন্তানের টিকা সম্পর্কে আপনাকে জিজ্ঞাসা কিছু করতে চাই।

Ask child's name and substitute "this child" with the name of the child in this section.
সন্তানের নাম জিজ্ঞাসা করুন এ□ং এই □িভাগে "এই শিশুর" স্থলে সন্তানের নাম □্য□হার করুন।

ID	QUESTION<br>প্রশ্ন	RESPONSE<br>উত্তর	GO TO<br>যান
201	Child #<br>**শিশুর নম্বর**	___ ___ ___ ___ ___ ___	
202	Child age<br>শিশুটির □য়স	_____ months / years *(circle)*<br>মাস/□ছর (□ৃত্ত আঁকুন)<br>☐88. Refused to answer উত্তর দিতে রাজি না	
203	Is the child present?<br>*আপনার শিশুটি এখানে উপস্থিত ?*	☐1. Yes হ্যাঁ<br>☐2. No না	
204	What is your relationship with the selected child?<br>নির্বাচিত শিশুর সঙ্গে আপনার সম্পর্ক কি?<br>*The respondent may consult with his/her spouse, children, neighbors, etc.*<br>উত্তরদাত তার স্বামী/ স্ত্রী, শিশু, প্রতি□েশী, ইত্যাদির সঙ্গে পরামর্শ করতে পার□েন	☐1. Mother মা<br>☐2. Father □া□া<br>☐3. Grandmother দাদী/ নানী<br>☐4. Grandfather দাদা/নানা<br>☐5. Aunt খালা/ ফুপু/ চাচী/কাকী/মামী<br>☐6. Uncle খালু/ফুপা/ চাচা/কাকা/মামা<br>☐7. Sister □োন<br>☐8. Brother ভাই<br>☐9. Other relative অন্যান্য অন আত্মীয়<br>☐10. Other অন্যান্য<br>☐88. Refused to answer উত্তর দিতে অস্বীকার	
205	Did this child arrive in Bangladesh together with the family?<br>শিশুটি কি পরি□ার সহ □াংলাদেশে এসেছিল ?	☐1. Yes হ্যাঁ --><br>☐2. No না<br>☐88. Refused to answer<br>উত্তর দিতে অস□ীকৃতি জানানো -----------><br>☐99. Don't know জানি না ------------------------->	Q207<br><br><br>Q207<br>Q207
206	If no, (the child arrived earlier or later), when did the child arrive in Bangladesh?<br>উত্তর না হলে (শিশুটি আগে/পরে আসলে) শিশুটি ক□ □াংলাদেশে এসেছিল ?<br>*Dates can be calculated based on Kurbani Eid (01 Sep 2017). Estimate the number of lunar months after Eid the child arrived.*	___ ___ lunar cycles after EID<br>ঈদের থথথথ চন্দ্রমাস পর<br>☐88. Refused to answer উত্তর দিতে অস্বীকার<br>☐99. Don't know জানি না	

	কুরবানির ঈদ (১ সেপ্টেম্বর, ২০১৭) থেকে তারিখ গণনা করা যেতে পারে। ঈদের পর থেকে চন্দ্রমাস গণনা করুন।		
207	Who takes the decision to vaccinate or not vaccinate this child?<br>আপনার শিশুর টিকা দেয়া / না দেয়া সিদ্ধান্ত কে নেয় ?<br><br>*Do not reach choices. Mark all that are mentioned.*<br>পছন্দগুলো বলবেন না। *যাদের নাম বলা হয় সবগুলো লিপিবদ্ধ করুন* ন।	☐1. Mother মা<br>☐2. Father বাবা<br>☐3. Sister বোন<br>☐4. Brother ভাই<br>☐5. Other female relative অন্য কোন মহিলা আত্মীয়<br>☐6. Other male relative অন্য কোন পুরুষ আত্মীয়<br>☐7. Other অন্যান্য<br>☐8. The child by himself or herself শিশু নিজেই<br>☐9. No one কেউ না<br>☐88. Refused to answer উত্তর দিতে অস্বীকার<br>☐99. Don't know জানি না	

If card is available, use date on vaccination card to answer questions. If no date is written on the card(s) or if card is missing, ask corresponding questions. For these questions, it may be helpful to use the event of Kurbani Eid, 01 Sep 2017, (or their arrival date if close to Kurbani Eid) to associate the first 2 campaigns (MR 1st round and OCV 1st round). As a reference, children under 2 years of age should have received injections in the thigh. Children 2 years and older should have received injections in the arm.

যদি কার্ড পাওয়া যায় তবে প্রশ্নগুলির উত্তর দেওয়ার জন্য টিকা কার্ডের তারিখ ব্যবহার করুন। যদি কোনও কার্ডে তারিখ লেখা না থাকে বা যদি কার্ড হারিয়ে যায়, তাহলে সংশ্লিষ্ট প্রশ্নগুলি জিজ্ঞাসা করুন।

এই প্রশ্নগুলি প্রথম দুটো টিকাদান ক্যাম্পেইন (এমআর ১ম রাউন্ড এবং ওসিভি ১ম রাউন্ড) এর সাথে সংযুক্ত করার জন্য কোরবানি ঈদ ০১ সেপ্টেম্বর ২০১৭, (অথবা তাদের আগমনের তারিখ যদি কোরবানি ঈদের কাছাকাছি হয়) ব্যবহার করা যেতে পারে।

একটি রেফারেন্স হিসাবে বলা যায়, ২ বছরের কম বয়সী শিশুদের ঊরুতে এবং ২ বছর এবং তার উপরের বয়সের শিশুদের বাহুতে ইনজেকশন পাওয়া উচিত।

	How many times did this child take diphtheria vaccine?<br>এই শিশুটি কতবার ডিপথেরিয়ার টিকা নিয়েছিল?<br><br>*This question is asked to guide the next few questions and will not be recorded.*<br>পরবর্তী কয়েকটি প্রশ্নের দিক নির্দেশনার এই প্রশ্নটি করা হবে কিন্তু রেকর্ড করা হবে না।		
208	About a month ago, there was a diphtheria vaccination campaign that included **one diphtheria injection** for older children. Did this child receive the injection? This child might have had **pain on touch** and maybe slight fever, which is normal. You may have received a **vaccination card** as well.<br><br>প্রায় এক মাস আগে, একটি ডিপথেরিয়া টিকাদান অভি□ান হয়েছিল, □খানে একটি ডিপথেরিয়া	☐1. Yes, confirmed by date in vaccination card ---><br>হ্যাঁ, টিকা কার্ডের তারিখ অনু□ায়ী নিশ্চিত<br>☐2. Yes, by recall -----------------------------------><br>হ্যাঁ, স্মরন দ্বারা<br>☐3. No না<br>☐88. Refused to answer উত্তর দিতে রাজি না ------><br>☐99. Don't know জানি না ------------------------->	Q307<br><br>Q307<br><br><br><br>Q309<br>Q309

	ইনজেকশন বয়স্ক শিশুদের দেয়া হয়েছিল।<br>আপনার শিশুটি কি সেখানে একটি ডিপথেরিয়া ইনজেকশন দেয়া হয়েছিল?<br><br>টিকাদান পরবর্তী সময়ে, সামান্য ব্যাথা অথবা সামান্য জ্বর হতে পারে, [illegible]া স্বাভাবিক। আপনি সেখানে একটি টিকাদান কার্ডও পেতে পারেন<br><br>*(10–29 March 2018:<br>Td, 2nd round for 7years–<15years)*<br><br>(মার্চ, ২০১৮: টিটেনাস-ডিপথেরিয়া ২য় রাউন্ড- ৭-১৫ বছর বয়সী শিশুর জন্য)		

209	What are the reasons why the child did not get vaccinated?<br>শিশুটির টিকা না পাওয়ার কারণগুলো কি ?<br><br>*Instructions:*<br>*The respondent should be prompted: 'Are there any other reasons?' until they have mentioned all reasons.*<br><br>*Do not read choices. Mark all that are mentioned.*<br><br>নির্দেশাবলী:<br>উত্তরদাতাকে অনুরোধ করা উচিত: 'অন্য কোন কারণ আছে কি না?'<br><br>পছন্দগুলি পড়বেন না । সেগুলো উল্লেখ করবে সেসব চিহ্নিত করুন।	☐1. Caregiver was unaware of the vaccination campaign<br>পরিচর্□াকারি টিকাদান ক্যাম্পেইন সম্পর্কে সচেতন ছিলনা<br>☐2. Caregiver thinks that child received all required vaccines<br>পরিচর্□াকারি মনে করে □ শিশুটি সব প্রয়োজনীয় টিকা গ্রহন করেছে<br>☐3. Caregiver does not think vaccines are necessary<br>পরিচর্□াকারি চান না □ শিশুটি টিকা গ্রহন করুক<br>☐4. Caregiver is fearful of vaccines or fearful of multiple injections<br>পরিচর্□াকারী অনেকগুলো ইনজেকশন এক সঙ্গে দিতে হবে, তাই ভয় পেয়েছিলেন<br>☐5. Caregiver or child is fearful about not going to heaven or being converted to Christianity<br>পরিচর্□াকারী জান্নাতে না □তে পারা অথবা খ্রীস্ট ধর্মে রূপান্তরিত হওয়ার ভয় পেয়েছেন<br>☐6. Female caregiver or child not comfortable leaving the house<br>মহিলা পরিচর্□াকারী অথবা শিশুটির পক্ষে ঘরটি ছেড়ে □াওয়া সম্ভবপর ছিল না<br>☐7. Female caregiver or child not comfortable taking vaccine from a male vaccinator<br>মহিলা পরিচর্□াকারী অথবা শিশুটির নিকট পুরুষ টিকাদানকারী গ্রহন□োগ্য ছিল না<br>☐8. The child got fever/swelling after last vaccination<br>শিশুটির শেষ টিকা গ্রহনের পর জ্বর / টিকার স্থানের ফুলে গিয়েছিল<br>☐9. No one was available to take the child to vaccination.<br>শিশুকে টিকা দিতে নিয়ে □াওয়ার জন্য কেউ ছিলনা<br>☐10. Child was not available (e.g., not at home, sick) at time of vaccination<br>শিশুটি বাসায় ছিল না / অসুস্থ ছিল<br>☐11. Child was afraid of needles/pain<br>শিশুটি ইনজেকশন/ ব্যাথার ভয় পেয়েছিল<br>☐12. Father or head of household does not allow<br>শিশুর পিতা / পরিবার প্রধান অনুমতি দেয়নি<br>☐13. Vaccinator treated caregiver or child poorly<br>টিকাদান কারী শিশু/ পরিচর্□াকারীর সাথে ভালো ব্যবহার করেননি<br>☐14. Other অন্যান্য<br>☐88. Refused to answer উত্তর দিতে অস্বীকার<br>☐99. Don't know জানি না	Q309
210	Who took the child to get vaccinated?<br>টিকা দিতে কে শিশুকে নিয়ে গিয়েছিল?	☐1. Mother মা<br>☐2. Father বাবা	

	Do not reach choices. Mark only one.<br>পছন্দগুলো পড়িবেন না, শুধুমাত্র একটিতে টিক চিহ্ন দিন।	☐3. Sister বোন<br>☐4. Brother fvB<br>☐5. Other female relative অন্য কোন মহিলা আত্মীয়<br>☐6. Other male relative অন্য কোন পুরুষ আত্মীয়<br>☐7. Neighbor/friend প্রতিবেশী / বন্ধু<br>☐8. Social mobilizer সমাজসেবী<br>☐9. Vaccinated at school স্কুলে টিকা দেয়া হয়েছিল<br>☐10. Vaccinated at madrassa মাদ্রাসায় টিকা দেয়া হয়েছিল<br>☐11. Child went by him/her self শিশু নিজে গিয়েছে<br>☐12. Other অন্যান্য ---------------------<br>☐88. Refused to answer উত্তর দিতে অস্বীকার<br>☐99. Don't know জানি না	
211	Before the last campaign, there was a diphtheria vaccination campaign that included **one diphtheria injection** for older children. Did this child receive the injection? This child might have had **pain on touch** and maybe slight fever, which is normal. You may have received a **vaccination card** as well.<br><br>গত টিকাদান অভিযান এর আগে আরও একটি ডিপথেরিয়া টিকাদান অভিযান হয়েছিল, যেখানে □য়স্ক শিশুদের একটি ডিপথেরিয়া ইনজেকশন দেয়া হয়েছিল।<br>আপনার শিশুটি কি সেখানে একটি ডিপথেরিয়া ইনজেকশন পেয়েছিল্ ?<br>টিকাদান পর□র্তী সময়ে, সামান্য □্যাথা অথ□া সামান্য জ্বর হতে পারে, যা স্বাভা□িক। আপনি সেখানে একটি টিকাদান কার্ডও পেতে পারেন<br><br>*(27 January–10 February 2018:*<br>*Td, 2nd round for 7years–<15years)*<br><br>(২৭ জানুয়ারী থেকে ১০ ফে□রুয়ারী, ২০১৮: ডিপথেরিয়া-টিটেনাস ২য় রাউন্ড- ৭ - ১৫ □ছর □য়সী শিশুর জন্য)	☐1. Yes, confirmed by date in vaccination card হ্যাঁ, টিকা কার্ডের তারিখ অনুযায়ী নিশ্চিত<br>☐2. Yes, by recall হ্যাঁ, স্মরন দ্বারা<br>☐3. No না<br>☐88. Refused to answer উত্তর দিতে রাজি না<br>☐99. Don't know জানি না	
212	And before that campaign, there was another diphtheria vaccination campaign that included **one diphtheria injection** for older children. Did this child receive the injection? This child might have had **pain on touch** and maybe slight fever, which is normal. You may have received a **vaccination card** as well.<br><br>এবং সেই টিকাদান অভিযান এর আগে আরও একটি ডিপথেরিয়া টিকাদান অভিযান হয়েছিল,	☐1. Yes, confirmed by date in vaccination card হ্যাঁ, টিকা কার্ডের তারিখ অনুযায়ী নিশ্চিত<br>☐2. Yes, by recall হ্যাঁ, স্মরন দ্বারা<br>☐3. No না<br>☐88. Refused to answer উত্তর দিতে রাজি না<br>☐99. Don't know জানি না	

	যেখানে বয়স্ক শিশুদের একটি ডিপথেরিয়া ইনজেকশন দেয়া হয়েছিল।<br>আপনার শিশুটি কি সেখানে একটি ডিপথেরিয়া ইনজেকশন পেয়েছিল্ ?<br>টিকাদান পরবর্তী সময়ে, সামান্য ব্যাথা অথবা সামান্য জ্বর হতে পারে, যা স্বাভাবিক। আপনি সেখানে একটি টিকাদান কার্ডও পেতে পারেন (২৭ জানুয়ারী থেকে ১০ ফেব্রুয়ারী, ২০১৮: ডিপথেরিয়া-টিটেনাস ১ম রাউন্ড- ৭ - ১৫ বছর বয়সী শিশুর জন্য)<br><br>*(12–31 December 2017:*<br>*Td, 1st round for 7years–<15years)*<br><br>(১২-৩১ ডিসেম্বর, ২০১৭: ডিপথেরিয়া-টিটেনাস ১ম রাউন্ড<br>৭ - ১৫ বছর বয়সী শিশুর জন্য)		
	How many times did this child take measles vaccine?<br>এই শিশুটি কতবার হামের টিকা নিয়েছে?<br><br>*This question is asked to guide the next few questions and will not be recorded.*<br>এই প্রশ্নটি পরবর্তী কয়েকটি প্রশ্নের দিক নির্দেশনার জন্য বলা হয়েছে এবং এটি রেকর্ড করা হবে না।		
213	Before the diphtheria vaccination campaign, there was a vaccination campaign that included **one injection for measles and NO oral drops**. Did this child receive the injection? You may have received a **vaccination card** as well.<br><br>ডিপথেরিয়া টিকাদান অভিযান এর আগে আরও একটি অভিযান হয়েছিল, যেখানে একটি হামের টিকার ইনজেকশন দেয়া হয়েছিল, কোনো পোলিও ড্রপ মুখে খাওয়ানো হযনি। আপনার শিশুটি কি টিকা পেয়েছিল? আপনি সেখানে একটি টিকাদান কার্ডও পেতে পারেন<br><br>*(18 November–05 December 2017:*<br>*MR, 2nd round for 6months–<15years)*<br><br>(১৮ নভেম্বর থেকে ৫ ডিসেম্বর, ২০১৭<br>এম আর ২য় রাউন্ড-<br>৬মাস - ১৫ বছর বয়সী শিশুর জন্য)	☐1. Yes, confirmed by date in vaccination card হ্যাঁ, টিকা কার্ডের তারিখ অনুযায়ী নিশ্চিত<br>☐2. Yes, by recall হ্যাঁ, স্মরন দ্বারা<br>☐3. No না<br>☐88. Refused to answer উত্তর দিতে রাজি না<br>☐99. Don't know জানি না	
214	There was another measles vaccination campaign a few weeks after Kurbani Eid that included cutting of the **Vitamin A red pill** and oral polio drops for young children and **one measles injection** for older	☐1. Yes, confirmed by date in vaccination card হ্যাঁ, টিকা কার্ডের তারিখ অনুযায়ী নিশ্চিত<br>☐2. Yes, by recall হ্যাঁ, স্মরন দ্বারা<br>☐3. No না	

	children. Did this child receive the measles injection? You may have received a **vaccination card** as well.<br><br>কুরবানির ঈদের কয়েক সপ্তাহ পরে আরও একটি হামের টিকাদান অভিযান হয়েছিল, যেখানে একটি ভিটামিন এ এর লাল পিল, একটি হামের টিকার ইনজেকশন দেয়া হয়েছিল ও পোলিও ড্রপ মুখে খাওয়ানো হয়েছিল ।<br>আপনার শিশুটি কি টিকাগুলো পেয়েছিল ?<br>আপনি সেখানে একটি টিকাদান কার্ডও পেতে পারেন<br><br>*(16 September–03 October 2017:*<br>*OPV only 0–<6months*<br>*OR MR+OPV+Vitamin A, 1st round for 6months–<5years*<br>*OR MR only for 5years–<15years)*<br><br>(১৬ সেপ্টেম্বর থেকে ০৩ অক্টোবর, ২০১৭:<br>ওপিভি-০ থেকে অনুর্ধ্ব ৬ মাস<br>ভিটামির এ+ এম আর + ওপিভি- ৬ মাস- অনুর্ধ্ব ৫ বছর বয়সী শিশুর জন্য<br>এম আর ১ম রাউন্ড ৫ বছর -অনুর্ধ্ব ১৫ বছর বয়সী শিশুর জন্য)	☐88. Refused to answer উত্তর দিতে রাজি না<br>☐99. Don't know জানি না	
215	After the Vitamin A / measles campaign, there was a vaccination campaign that included **<u>oral cholera vaccine in a small bottle for both adults and children</u>** (from 1 year and above). Did this child receive the oral vaccine? You may have received **soap** as well.<br><br>ভিটামিন এ /হামের টিকাদান ক্যাম্পেইনের পরে, আরেকটি টিকাদান ক্যাম্পেইন হয়েছিল যার মধ্যে অন্তর্ভুক্ত ছিল একটি ছোট বোতল যা ছিল বয়স্ক ও শিশুদের উভয়ের জন্য (১বছর এবং তারও বেশি)। এই শিশুটিকে কি মৌখিক টিকা দেওয়া হয়েছিল? আপনি একটি সাবান ও পেয়ে থাকতে পারেন।<br><br>*(10–18 October 2017:*<br>*OCV, 1st round for ≥1year)*<br><br>(১০-১৮ অক্টোবর ২০১৭: ওসিভি প্রথম রাউন্ড) > ১ বছর)	☐1. Yes হ্যাঁ<br>☐2. No না<br>☐88. Refused to answer উত্তর দিতে রাজি না<br>☐99. Don't know জানি না	
216	When you and your family entered Bangladesh, did this child take any	☐1. Yes, confirmed by date in vaccination card হ্যাঁ, টিকা কার্ডের তারিখ অনুযায়ী নিশ্চিত<br>☐2. Yes, by recall হ্যাঁ, স্মরন দ্বারা	

	vaccination at the border, either injections or oral polio drops?<br>যখন আপনি এবং আপনার পরিবার বাংলাদেশে ঢুকলেন, তখন এই শিশুটিকে কি সীমান্তে কোনও টিকা - ইনজেকশন বা মৌখিক পোলিও ড্রপ দেওয়া হয়েছিল?	☐3. No না<br>☐88. Refused to answer উত্তর দিতে রাজি না<br>☐99. Don't know জানি না	
217	Can you share any additional vaccination cards or medical records related to this child?<br>আপনি কি এই শিশুর সাথে সম্পর্কিত কোনও অতিরিক্ত টিকাদান কার্ড বা মেডিকেল রেকর্ড দেখাতে পারেন?<br><br>*If there are any additional vaccination cards that do not correspond to campaign doses, vaccination at the border or vaccination for being a diphtheria contact, indicate which vaccines were received.*<br>যদি কোনো অতিরিক্ত টিকাদান কার্ড থাকে যা ক্যাম্পেইন ডোজ, সীমান্তে টিকা বা ডিপথেরিয়ার কারনে টিকার সাথে সম্পর্কিত নয় তাহলে, কোন টিকা তা নির্দেশ করুন।	☐1. OPV, confirmed by date in vaccination card<br>ওপিভি, টিকা কার্ডের তারিখ অনুযায়ী নিশ্চিত<br>☐2. PCV, confirmed by date in vaccination card<br>পিসিভি টিকা কার্ডের তারিখ দ্বারা নিশ্চিত<br>☐3. Penta, confirmed by date in vaccination card<br>পেন্টা টিকা কার্ডের তারিখ অনুযায়ী নিশ্চিত<br>☐4. Td, confirmed by date in vaccination card<br>টিডি, টিকা কার্ডের তারিখ দ্বারা নিশ্চিত<br>☐5. MR, confirmed by date in vaccination card<br>এম.আর.টিকা কার্ডের তারিখ অনুযায়ী নিশ্চিত<br>☐6. BCG, confirmed by date in vaccination card<br>বিসিজি, টিকাদান কার্ড থেকে নিশ্চিত<br>☐7. IPV, confirmed by date in vaccination card<br>আইপিভি, টিকাদান কার্ড থেকে নিশ্চিত<br>☐8. Other অন্যান্য<br>☐9. No other vaccination card available<br>অন্য কোন টিকা কার্ড পাওয়া যায় না	
218	When this child lived in Myanmar, did this child ever take polio vaccine or oral polio drops. Polio is a vaccine given by dropping liquid into a child's mouth. Oral polio drops are different from Vitamin A drops, which usually are red or blue pills.<br>যখন এই শিশুটি মায়ানমারে বসবাস করত, তখন এই শিশুটি কি কখনো পোলিও টিকা বা মৌখিক পোলিও ড্রপ গ্রহন করেছে? পোলিও একটি তরল টিকা যা শিশুর মুখের মধ্যে ড্রপ দ্বারা দেওয়া হয়।	☐1. Yes, confirmed by date in vaccination card<br>হ্যাঁ, টিকা কার্ডের তারিখ অনুযায়ী নিশ্চিত<br>☐2. Yes, by recall হ্যাঁ, স্মরন দ্বারা<br>☐3. No না<br>☐88. Refused to answer উত্তর দিতে রাজি না<br>☐99. Don't know জানি না	
219	When this child lived in Myanmar, did this child ever take any vaccination through injection?<br>যখন এই শিশু মায়ানমারে বসবাস করত, তখন কি এই শিশুটি ইনজেকশন এর মাধ্যমে কোনো টিকা নিয়েছে ?	☐1. Yes, confirmed by date in vaccination card<br>হ্যাঁ, টিকা কার্ডের তারিখ অনুযায়ী নিশ্চিত<br>☐2. Yes, by recall হ্যাঁ, স্মরন দ্বারা<br>☐3. No না<br>☐88. Refused to answer উত্তর দিতে রাজি না<br>☐99. Don't know জানি না	
220	*Does the child have a BCG scar? Check the child's upper right and left arms. (Do not ask the caregiver.)*<br>এই শিশুটির কি বিসিজি স্কার আছে? শিশুর ডান এবং বাম বাহুর উপরে পরীক্ষা করুন। (পরিচর্যাকারিকে জিজ্ঞাসা করবেন না।)	☐1. Yes হ্যাঁ<br>☐2. No না<br>☐3. Not sure if scar is from BCG<br>নিশ্চিত না স্কারটি বিসিজির কিনা<br>☐99. Child not available শিশুটি উপস্থিত নাই	
221	In the past two weeks, has this child had a fever without rash?<br>বিগত ২ সপ্তাহে কি শিশুর র‍্যাশ ছাড়া জ্বর হয়েছিলো ?	☐1. Yes হ্যাঁ<br>☐2. No না<br>☐88. Refused to answer উত্তর দিতে রাজি না<br>☐99. Don't know জানি না	
222	After arrival in Bangladesh, has this child had measles?	☐1. Yes, confirmed by health facility document<br>হ্যাঁ, স্বাস্থ্য কেন্দ্রের দলিল অনুযায়ী নিশ্চিত	

	বাংলাদেশে আগমনের পর, শিশুর কি কখনো হাম হয়েছিলো ?	☐2. Yes, verbal report that the child was diagnosed at a clinic<br>হ্যাঁ, কোনো ক্লিনিকে মৌখিকভাবে নিশ্চিত<br>☐3. Yes, verbal report that the child was diagnosed by a local healer<br>হ্যাঁ, কোনো স্থানীয় পল্লী চিকিসক কর্তৃক মৌখিকভাবে নিশ্চিত<br>☐4. Yes, verbal report but did not seek diagnosis<br>হ্যাঁ, মৌখিক রিপোর্ট কিন্তু ওই রোগের জন্য যায়নি<br>☐5. No না<br>☐88. Refused to answer উত্তর দিতে রাজি না<br>☐99. Don't know জানি না	
223	After arrival in Bangladesh, has this child had diphtheria?<br>বাংলাদেশে আগমনের পর, শিশুর কি কখনো ডিপথেরিয়া হয়েছিলো ?	☐1. Yes, confirmed by health facility document<br>হ্যাঁ, স্বাস্থ্য কেন্দ্রের দলিল অনুযায়ী নিশ্চিত<br>☐2. Yes, verbal report that the child was diagnosed at a clinic<br>হ্যাঁ, কোনো ক্লিনিকে মৌখিকভাবে নিশ্চিত<br>☐3. Yes, verbal report that the child was diagnosed by a local healer<br>হ্যাঁ, কোনো স্থানীয় পল্লী চিকিসক কর্তৃক মৌখিকভাবে নিশ্চিত<br>☐4. Yes, verbal report but did not seek diagnosis<br>হ্যাঁ, মৌখিক রিপোর্ট কিন্তু ওই রোগের ক্লিনিকে জন্য যায়নি<br>☐5. No না<br>☐88. Refused to answer উত্তর দিতে রাজি না<br>☐99. Don't know জানি না	

END OF QUESTIONNAIRE

ধন্যবাদ জানিয়ে সাক্ষাতকারটি শেষ করুন।

INSERT Bangla of above end instruction.
